# Supplementary material for: Nanoparticle elasticity affects systemic circulation lifetime by modulating adsorption of apolipoprotein A-I in corona formation
Source: Nat Commun. 2022 Jul 16;13:4137. doi: 10.1038/s41467-022-31882-4 (PMC9288426; doi:10.1038/s41467-022-31882-4)
Supplement: Supplementary file 1 — Supplementary Information [file 41467_2022_31882_MOESM1_ESM.pdf]

Supplementary Information

Nanoparticle Elasticity Affects Systemic Circulation Lifetime by Modulating Adsorption of Apolipoprotein A-I in Corona Formation

Mingyang Li[1,2,3], Xinyang Jin[1,2,3], Tao Liu[1,2,3], Feng Fan[1,2,3], Feng Gao[1,2,3], Shuang Chai[1,2,3] and Lihua Yang*[1,2,3]

[1]Hefei National Research Center for Physical Sciences at the Microscale, University of Science and Technology of China, Hefei, Anhui 230026 China. [2]CAS Key Laboratory of Soft Matter Chemistry, University of Science and Technology of China, Hefei, Anhui 230026 China. [3]School of Chemistry and Materials Science, University of Science and Technology of China, Hefei, Anhui 230026 China.

*Corresponding author: (L.Y.) lhyang@ustc.edu.cn

ADDITIONAL RESULTS AND DISCUSSION

1. **Nanogel@lipid particles have a crosslinked nanogel core successfully encapsulated within a lipid shell.**

To prepare a nanogel@lipid, the hydrogel precursor solution was preloaded within a liposome (d > 100 nm) and then subjected to photo-polymerization to form a crosslinked nanogel as the core. To confirm the formation of a nanogel core inside a liposome in a nanogel@lipid, we treated our nanogel@lipid particles with Triton X-100, a detergent good at removing lipids from a lipid bilayer into micelles with sizes of ~10 nm[1] (Supplementary Figure 2a), with DOPC : DSPE-PEG_{2000} = 90 : 10 liposome included as a reference. The ability of Triton X-100 to remove the DOPC and DSPE-PEG_{2000} lipids into micelles was confirmed by the observations that, after treatment with Triton X-100, dispersion of DOPC : DSPE-PEG_{2000} = 90 : 10 liposome exhibited both the disappearance of particles with sizes of >100 nm and the appearance of particles with sizes of ~10 nm (Supplementary Figure 2b). In contrast, for all our nanogel@lipid particle dispersions, similar treatment with Triton X-100 did not lead to disappearance of particles with sizes of >100 nm (Supplementary Figure 2c), indicative of retention of the core nanogel nanoparticles with sizes of >100nm, suggesting successfully formation of a nanogel core within a lipid bilayer shell as expected; instead, Triton X-100 treatment made nanogel@lipid dispersions exhibit the appearance of particles with sizes of ~10 nm, due to micelle formation by removed lipids and Triton X-100. Moreover, using 75kPa@lipid as a representative for nanogel@lipid particles, we found that Triton X-100 treatment barely affected the Tyndall effect in 75kPa@lipid dispersion but significantly reduced that in the dispersion of DOPC : DSPE-PEG_{2000} = 90 : 10 liposome (Supplementary Figure 2c). Collectively, these observations suggest that the expected nanogel@lipid nanoparticles were successfully prepared, in which a crosslinked nanogel core was successfully encapsulated within a lipid shell.

2. PLGA@lipid and PLGA@lec nanoparticles were successfully prepared.

Coating the PLGA nanoparticle with a lipid bilayer was accomplished by sonicating the mixture of PLGA and liposome (DOPC: DSPE-PEG_{2000} = 90:10) or lecithin liposome (lec) (mass ratio of lipids to PLGA = 1:1) in a water bath sonicator for 5 min, followed by centrifugation to remove excess liposome, which yielded the as-expected lipid bilayer-coated nanoparticle (namely, PLGA@lipid or PLGA@lec).

Under TEM, PLGA@lipid (Supplementary Figure 4a) and PLGA@lec (Supplementary Figure 18a) nanoparticles appeared spherical, with a core-shell structure. After coating with liposomes composed of DOPC: DSPE-PEG_{2000} = 90:10, the average hydrodynamic diameters of PLGA nanoparticles increased relatively by 25.7 nm, consistent with prior reports that showed relative increases of 20-30 nm in average size for nanoparticles after coating them with PEGylated lipid bilayer[2-5]. Similarly, after coating with liposomes composed of lecithin, the average hydrodynamic diameters of PLGA nanoparticles increased relatively by 26.4 nm, consistent with prior reports that demonstrated relative increases of ~20 - ~22 nm in size for nanoparticles after coating with lecithin lipid bilayer [6, 7]. Meanwhile, after the coating procedure, zeta-potentials of the resulting PLGA@lipid and PLGA@lec particles (Supplementary Figure 4c and Supplementary Figure 18) became appreciably different from those of bare PLDA particles but close to those of the DOPC: DSPE-PEG_{2000} = 90:10 and lecithin liposomes, respectively. Collectively, these results indicate that PLGA@lipid and PLGA@lec nanoparticles were successfully prepared.

3. The stability of our nanoparticles.

The lipid bilayer coating is very stable for our nanogel@lipid and PLGA@lipid nanoparticles. To examine the stability of the lipid bilayer over nanoparticle, we used 188kPa@lipid and 700kPa@lipid as the representatives for our model nanoparticles and compared their morphologies under Cryo-electron microscopy (Cryo-EM) before and after treatment that simulates the conditions our model nanoparticles were to encounter both *in vitro* and *in vivo*.

To construct the control nanoparticles resulted from complete peel-off of the lipid bilayers from our model nanoparticles, we treated 188kPa@lipid and 700kPa@lipid nanoparticles with Triton X-100, as treating lipid bilayer-coated nanoparticles with Triton X-100 is a demonstrated method to peel off their lipid bilayer coating[1], and then characterized the morphology of the as-treated nanoparticles under Cryo-EM. Moreover, to simulate the conditions our model nanoparticles were to encounter in the *in vitro* cell studies and in the *in vivo* animal studies and to examine whether the lipid bilayer coatings over our model nanoparticles were stable under those conditions, we incubated our nanoparticles in fetal bovine serum (FBS)-supplemented phosphate buffered saline (PBS) (v./v., 50%) for 7 consecutive days, separated the particles from supernatant *via* centrifugation at 150,000 g at 4 ºC for 1 h, and collected (after discarding the resulting supernatant) the resulting pellet (*i.e.*, the as-incubated particles) by washing with PBS for two times, redispersed the as-collected pellet into PBS, and then characterized the morphologies of the as-incubated nanoparticles under Cryo-EM.

Our results (Supplementary Figure 6) reveal that, on the 1st day after being dispersed into PBS, our pristine 188kPa@lipid and 700kPa@lipid nanoparticles (left column) both exhibited an intact core-shell structure under Cryo-EM, in which a thin shell (thickness < 10 nm) (as indicated by white arrow) is clearly observed over the outermost surface of a nanoparticle of slightly lower electron density contrast (Supplementary Figure 6, left column). In stark contrast, the particles after Triton X-100 treatment (Supplementary Figure 6, middle column) completely lost the core-shell structure characteristic of their pristine precursors (*i.e.*, 188kPa@lipid and 700kPa@lipid particles) but instead appeared to be nanospheres with near uniform electron density contrast yet sizes comparable with those of their pristine precursors. In addition to these nanospheres, there emerged many tiny particles of much smaller sizes (<10 nm) (indicated by red arrow), which are likely attributable to lipid micelles formed by the lipids after their peel-off from the nanoparticle cores by Triton X-100, consistent with what was observed in the dynamic light scattering experiments (Supplementary Figure 2). Interestingly, the resulting particles after 7-day incubation in FBS-supplemented PBS (Supplementary Figure 6, right column) exhibited a core-shell structure, in which a thin shell (thickness < 10 nm) (as indicated by white

arrow) is clearly observed over the outermost surface of a nanoparticle of slightly lower electron density contrast, as did their pristine precursors (*i.e.*, 188kPa@lipid and 700kPa@lipid particles) (Supplementary Figure 6, left column). Collectively, these observations indicate that 188kPa@lipid and 700kPa@lipid particles both retained their characteristic core-shell structure even after 7-day incubation in FBS-supplemented PBS, rather than losing their lipid bilayer shell as observed with the same particles but after Triton X-100 treatment, suggesting that the lipid bilayer coating over our model nanoparticles is stable.

4. Reasons why 45 kPa was adopted as the elasticity of our empty liposome.

We adopted a previously reported elasticity of 45 kPa for our PEGylated liposome DOPC : DSPE-PEG_{2000} = 90 : 10), for reasons as follow. Firstly, we found quite some studies that have reported the elasticity for liposomes of differing compositions Supplementary Table 4) but were puzzled by that their reported Young's moduli differed strikingly (either <50 kPa or ≥500 kPa) depending on the specific lipid composition and the specific methods and conditions used to measure the Young's modulus. Secondly, a cell is much more complex than a liposome, despite that liposomes are widely used as simplified models for cells especially in biochemistry and biophysics studies; although lipid bilayer is the major membrane component for both liposomes and cells, a cell has other crucial components/parts like membrane proteins in its cellular membrane, a cytoskeleton network, and a crowded cytosol containing diverse organelles. Therefore, the elasticity of a liposome should be on the same or similar order-of-magnitude as that of a cell. Therefore, we summarized the reported elastic moduli for different cells (Supplementary Table 5) and found that they are unanimously <50 kPa. Therefore, we think, among the reported Young's moduli for liposomes (Supplementary Table 4), those of <500 kPa should be more likely for empty liposomes composed of glycerophospholipids above their phase transition temperatures such as our empty PEGylated liposome.

5. The slight difference in zeta-potential readings for our model nanoparticles does not mean difference in surface chemistry.

For a core-shell structured particle, the core of the particle may partially screen the charges on the outermost layer of the particle (*i.e.*, the outer-surface of its shell) and consequently affect the ion adsorption within the electrical double layer surrounding the particle — which in return determines its zeta-potential. In another word, core-shell structured particles of a same shell (*i.e.*, same surface chemistry) but different cores may exhibit different zeta-potentials (Supplementary Figure 5) due to the contribution of the particle core. Indeed, supportive evidences to this are found in our literature research on the reported zeta-potentials of lipid or cellular membrane-coated nanoparticles (Supplementary Table 6); as our model nanoparticles in this work are liposomes and lipid bilayer-coated particles, we confined our literature research to lipid or cellular membrane-coated nanoparticles.

Our literature research results (Supplementary Table 6) revealed that none of the lipid or cellular membrane-coated nanoparticles exhibited a same zeta-potential as did its corresponding shell (*i.e.*, the membrane vesicle used to coat the nanoparticle of interest), despite of their similarity in surface chemistry. Of note, this conclusion retains despite that the lipid or cellular membrane-coated nanoparticles we found contain cores differing significantly in materials (inorganic *versus* organic *versus* bacterial) or shells of distinct compositions (synthetic lipid bilayer of distinct compositions *versus* natural cellular membranes of distinct origins). Moreover, zeta-potential even differs between membrane-coated nanoparticles of a same membrane shell but slightly differing cores [8-10]; this is the case no matter whether the cores are inorganic SiO_2 nanoparticles[10], polymeric PLA (polylactic acid) nanoparticles[8], or bacterial cells[9]. Clearly, for a core-shell structured nanoparticle, its shell is not the sole factor that determines its zeta-potential; on this aspect, its core matters as well. In another word, that core-shell structured nanoparticles exhibit different zeta-potentials does not necessarily suggest that these particles must differ in surface chemistry.

Back to our model nanoparticles in this work, they are core-shell structured

nanoparticles, in which the shell is a lipid bilayer while the core varies from water over soft hydrogels to PLGA (poly(lactic-co-glycolic acid)) nano-micelle. In lipid relevant research fields, it is a widely-acknowledged practice that the chemistry of a liposome or lipid bilayer is determined by the lipid composition used for preparing the liposome or lipid bilayer[11-14]. Our model nanoparticles (excepting PLGA@lipid and PLGA@lithicin) were prepared by following the standard rehydration technique for liposome preparation except that a slight modification was incorporated for hydrogel@lipid nanoparticles; the liposomes were prepared by following the standard rehydration technique for liposome preparation, and the hydrogel@lipid nanoparticles were prepared through a similar rehydration technique except having replaced ultra-pure water for rehydration with hydrogel precursor solutions of differing monomer to cross-linker relative ratios - which after extrusion and ultra-violet light irradiation in subsequent steps provided lipid bilayer-enclosed hydrogel nanoparticles of differing elasticity. Through this procedure, the resulting liposome (no matter whether their interior content is pure water or a hydrogel precursor solution) offers a self-enclosing lipid bilayer (*i.e.*, the shell) whose chemistry is determined by the lipid composition used for preparing the dehydrated lipid thin film, which after rehydration offers the lipid bilayer shell for the liposome and the hydrogel@lipid nanoparticles. In this work, we used two sets of model nanoparticles, with one set having a lipid bilayer shell with a composition of DOPC: DSPE-PEG_{2000} = 90: 10 (mass ratio) while another set having a lipid bilayer shell composed of lethicin alone (*i.e.*, lethicin at a mass ratio of 100%). Within each set of our model nanoparticles, we correspondingly fixed the lipid composition for preparing the dehydrated lipid thin film either at DOPC: DSPE-PEG_{2000} = 90: 10 or lethicin alone. The two stiffest particles, PLGA@lipid and PLGA @lethicin, were prepared by coating a preformed PLGA nano-micelle with a liposome (composed of either DSPE-PEG_{2000} = 90: 10 or 100% lethicin), and coating a liposome over a nanoparticle is virtually a process that transfers the self-enclosing lipid bilayer of the liposome as a lipid bilayer shell over the particle[15, 16] and the chemistry of the resulting lipid bilayer shell is determined by the lipid composition of the liposome[17, 18]. Therefore, it is reasonable for us to expect that, within a given set of

our model particles, all particles should be the same in surface chemistry. Specifically, for all our DOPC: DSPE-PEG_{2000} = 90: 10 bilayer-coated nanoparticles, the surface PEG density, which is determined by the relative content of DSPE-PEG_{2000}, should be similar.

Of course, to unveil the effects of elasticity on nanoparticle fate in physiological environment, it is necessary to dissect the effects of elasticity from those of other physiochemical factors like surface charge and surface PEG density. As stated above, within each given set of our model nanoparticle, the particles are reasonably expected to be same or similar in surface chemistry, which includes surface charge and surface PEG density if PEGylated lipid was involved. Based on analysis above, we reasonably expect that the reason why our DOPC: DSPE-PEG_{2000} = 90: 10 bilayer-coated nanoparticles exhibited slightly different readings in zeta-potential (Figure 1e) must arise because of their significantly differing cores, which ranges from ultra-pure water (for the liposome) over hydrogel cores of differing elasticity (for the hdyrogel@lipid particles) to PLGA nano-micelles (for the PLGA@lipid).

Of note, the zeta-potentials of our DOPC: DSPE-PEG_{2000} = 90: 10 bilayer-coated nanoparticles are unanimously negative (Figure 1e). Using polystyrene latex particles that are increasingly negative in surface charge density as model nanoparticles, a previous study has found that increasing nanoparticle surface charge density increases the total amount of adsorbed proteins but imposes negligible effects on the qualitative and quantitative composition of the adsorbed protein pattern[19]. A similar trend was observed in a review paper[20] which summarized the influence of nanoparticle surface charge on protein corona for anionic polystyrene nanoparticles (Supplementary Table 7). In contrast, when the zeta-potentials of particles are changed from negative to positive, the adsorbed protein pattern will be significantly changed[21, 22]. Therefore, for nanoparticles that differ only in zeta-potential, it is the sign of their zeta-potentials, rather than the absolute values of their zeta-potentials, that significantly affect their corona protein compositions.

6. Shorter circulation lifetime for liposome than lipid bilayer-coated hard nanoparticles in prior reports.

Compared with particles from Region I and Region III, those from Region II - which included not only liposomes but also very soft hydrogel particles (Supplementary Table 13) - exhibited the shortest blood clearance half-lives (Figure 2c). Supportive evidences for our conclusion that particles from Region II have shorter circulation lifetimes than particles from Region III were found in previous studies that, though not examining how particle elasticity affects particle blood circulation lifetime, happened to have reported the circulation lifetime of particles whose elasticity values belong to Regions II and III (Supplementary Table 10). One research article[23] reported that a lipid bilayer-coated mesoporous silica nanoparticle exhibited a blood clearance half-life of 11 h, which is appreciably longer than that (8.7 h) of the corresponding empty liposome enclosed by the same lipid bilayer (DSPC/cholesterol/DSPE-PEG_{2000}). Another article[24] reported that the blood circulation half-life of lipid bilayer-coated mesoporous silica nanoparticle was ~9.6 h as compared to ~3.3 h for the corresponding empty liposome (DSPC/cholesterol/DSPE-PEG_{2000}). Clearly, the observations in these articles suggest that softer particles do not necessarily exhibit longer blood circulation lifetimes than their stiffer counterparts, which is consistent with our conclusion in this work.

Although few reports compared side-by-side the blood circulation lifetime of liposomes *versus* lipid bilayer-coated nanoparticles in a same study, many works had separately reported the blood circulation lifetimes of liposomes and lipid membrane-coated stiff nanoparticles. We collected the reported blood circulation lifetimes of liposomes and lipid membrane-coated stiff particles that are scattered in different articles and plotted these data into one single plot (Supplementary Figure 9), which again supports our conclusion that particles from Region II have shorter blood circulation lifetime than those from Region III (Figure 2c).

7. LC-MS analysis on protein corona.

LC-MS analysis not only identified the adsorbed proteins in corona of a nanoparticle but also quantified their relative abundances. When sorted by molecular weight (Figure 3c), the corona proteins were enriched (>80%) in proteins of <60 kDa in all nanoparticles, suggesting preference for smaller plasma proteins, consistent with prior reports[25, 26]. When sorted by isoelectric point (pI), the corona proteins were enriched (40-70%) with negatively charged proteins (pI < 7) (Figure 3d) despite of the negative surface charges of our nanoparticles, consistent with a prior study on negatively charged silica nanoparticles[27] yet contrasting significantly with some recent reports[28-31] that show enrichment of positively charged proteins on negatively charged nanoparticles. When sorted by biological functions (Figure 3e), corona proteins were enriched in lipoproteins and complement proteins. Of note, no matter how adsorbed proteins were classified, protein compositions varied appreciably yet non-monotonically depending on nanoparticle elasticity (Figure 3c-e). Collectively, these results suggest that nanoparticle elasticity affects protein corona in both the total amount and protein pattern.

8. Lack of correlation between the relative abundance in protein corona and the protein content in blood.

Intuitively, it is easier for proteins with higher relative abundances in blood to get in contact with a particle and then adsorb onto the particle's surface, thereby leading to higher relative abundance in corona. Nevertheless, this is not the case for all our nanoparticles, for which no correlation was found between the relative abundance content of a protein in corona and its corresponding abundance in blood (Figure 3f), consistent with prior reports[32, 33]. For example, albumin, one of the most abundant proteins in blood, presented with a relative abundance of only 1-2% in corona on all our particles. This phenomenon may be ascribed to nanoparticles' physicochemical properties, which may have strongly impacted a nanoparticle's

interactions with plasma proteins and, as a result, resulted in selective enrichment of certain plasma proteins in the particle's corona.

9. Clusterin and pentaxin have their relative abundances in corona strongly correlated with the uptake by adherent and suspended macrophage cells, respectively.

Although a clear dependence on nanoparticle elasticity was not observed for nanoparticles' uptake by adherent cells (Figure 2i) and whether a clear dependence on nanoparticle elasticity ever exists for nanoparticles' uptake by suspended cells is largely unknown (Figure 2k), we still examined whether there exist certain corona proteins whose relative abundances in corona strongly correlate with cellular uptake efficiency, by calculating Pearson's correlation coefficient r between the relative abundance of a major corona protein (Figure 3f-h) and cellular uptake efficiency (Figure 2e-h for adherent cells, and Figure 2k for suspended cells) for our model nanoparticles. We started with adherent cells in plasma-supplemented medium (DMEM) (Figure 2g-h), an *in vitro* simulation of the interactions between nanoparticles and resident phagocytes *in vivo*. Our calculations on Pearson's r revealed that, among major corona proteins, clusterin is the only one that consistently exhibited a strong correlation between relative abundance in corona and uptake efficiency by adherent macrophage cells (both RAW264.7 and Ana-1) (Figure 2g-h), and its Pearson's r of >0.6 suggests clusterin as an opsonin for adherent cells, as observed differently before with lipid-free polymeric hard nanoparticles[34]. This may due to the low abundance (< 1%) in all nanoparticles except PLGA@lipid, the low abundance is not enough to contribute to the cellular uptake. We next considered suspended Ana-1 macrophage cells in mouse plasma-supplemented medium (RPMI 1640) (Figure 2k), an *in vitro* simulation of the interactions between nanoparticles and circulating phagocytes *in vivo*, and carried out similar calculations on Pearson's r (Supplementary Figure 17). Our results revealed pentaxin as the only major corona

protein whose relative abundance in corona exhibited a strong correlation with the uptake by suspended Ana-1 cells (Supplementary Figure 17c), with a Pearson's r of <-0.6 consistently with both trials; still, it should be noted that, in coronas of all our nanoparticles, pentaxin presents at very low relative abundance (Supplementary Figure 17), which may limit its function in cellular uptake process.

10. Information on ApoA1 from human source and that from mouse source.

In our ITC experiments (Figure 4a-b) and also cell studies on how ApoA1 supplementation affects nanoparticles' cellular uptake (Figure 4c-d), we used ApoA1 from human source, rather than that from mouse source out of the following two reasons. Firstly, ApoA1 from both human and mouse sources have similar molecular weight (~28 kDa)[35, 36], very similar sequences[37] and almost the same physiological functions *in vivo* —which participate in many similar biological processes (like cholesterol metabolism, lipid metabolism and transport and so on) by comparing on the website of Uniprot (https://www.uniprot.org), which is a freely accessible database of protein sequence and functional information. Secondly, a prior study[34] on protein corona examined the effects of clusterin on cellular uptake by incubating nanoparticles with murine cells in the presence *versus* absence of clusterin from human source, suggesting human source as acceptable substitution for mouse source for certain plasma proteins.

Supplementary Tables

Supplementary Table 1. Reported effects of nanoparticle elasticity on blood circulation clearance lifetime.

Particle	Size (nm)	Elasticity (kPa)	$t_{1/2}$ circulation clearance half-life (h)	Ref.
PEG-based hydrogel spheres	~200	10 and 3000	61.4 and 9.7	38
Zwitterionic nanogels	~120	180, 260, 580, 870, and 1350	19.6, 15, 11.8, 10.2, and 9.1	39
PLGA-PEG-lipid disks	1000 × 400	1.3 and 15	Longer circulation for softer one	40
Red Blood Cell Mimics particles	(5200–5900) × (1220–1540)	7.8, 16.9, 39.6, and 63.9	93.3, 7.12, 5.12, and 2.88	41

Supplementary Table 2. Reported effects of nanoparticle elasticity on cellular uptake efficiency of adherently cultured cells.

Particle	Size (nm)	Elasticity (kPa)	Cell-lines	Serum supplemented in culture medium	Ref.
**Higher uptake for stiff nanoparticles**					
PEG-based hydrogel spheres	~200	10 and 3000	J774	10% FBS	38
Silica nanoparticles	~150	704–9700000	RAW264.7	10% FBS	42
PLGA-PEG-lipid disks	1000 × 400	1.3 and 15	J774	10% FBS	40
Polyacrylamide beads	1000–6000	No report	Bone-marrow-derived macrophages	no report	43
TA/PVPON capsules and the corresponding cores coated with TA/PVPON	2000–3000	Sphere: 4300 and >104000<br>Cube: 610 and >104000	J774	10% FBS	44
**Higher uptake for soft nanoparticles**					
Organosilica nanocapsule and nanoparticle	~300	3950 and 251000	RAW264.7	no report	45
**Higher uptake for nanoparticles with intermediate elasticity**					
DEA-HEMA hydrogel spheres	~170	18, 35, 136, and 211	RAW264.7	10% FBS	46
PGA-CpG nanocapsule	~1300	2.5–22.5	PBMCs cell	10% FBS	47
**Negligible influence of nanoparticle elasticity on cellular uptake**					
PEGMA/GMA cylindrical polymer brushes	~1200	No report	RAW264.7	10% FBS	48

Supplementary Table 3. Compositions of the hydrogel precursor solutions used for preparing nanogel@lipid particles.

Sample	Monomer (w/v)	Cross-linker (w/v)	Photoinitiator (w/v)
75kPa@lipid	10%	1%	0.1%
100kPa@lipid	10%	3%	0.1%
188kPa@lipid	10%	5%	0.1%
350kPa@lipid	20%	1%	0.1%
700kPa@lipid	20%	5%	0.1%
1400kPa@lipid	40%	3%	0.1%
1700kPa@lipid	40%	5%	0.1%

Supplementary Table 4. The values for Young's modulus of empty liposomes.

Composition	Young's Modulus (kPa)	Ref.
DSPC/CHOL/DSPE-PEG_{2000}	40	49
DOPC	45	50
Egg-PC	1970	51
DOPC	13000	52
DOTAP/glutaryl PE/Tween 80	650	53
DSPC/DSPG/CHOL	1500	54
DPPC/DPPG/CHOL	1200	
DOPC/DOPG	500	

Supplementary Table 5. The values for Young's modulus of cells.

Cell	Young's modulus (kPa)	Ref.
Platelets	1-50	55
Fibroblasts	5	56
Neutrophils	1.548	57
RBCs	26 ± 7	58
Hepatoma cells	34.137	59
Hu609	12.9 ± 4.8	60
BC3726	1.4 ± 0.7	
Hu456	0.4 ± 0.3	
Stimulated macrophages	0.29	61
Resting macrophages	0.83	
Living undifferentiated adipose-derived stem cells	1.27	62
Dead undifferentiated adipose-derived stem cells	18.61	

Supplementary Table 6. The summary of the surface charge core-shell nanoparticles.

Composition		Zeta-potential (mV)			Ref.
core	shell	core	core-shell NP	shell	
polystyrene NP [a]	DPPC	-58	-40	-3	63
	DPTAP		22	62	
AuNR [b]	DPPC/DPPG	54.1	27.4	-19.2	64
AuNP	DPPC/DPTAP	-27.33	-9.87	13.78	
ZnO NP	DOPC	26	1.3	-15	65
Fe_3O_4 NP	Cancer-erythrocyte hybrid membrane	-31.23	-24.54	-19.28	66
PCL-PEG [c] NP	Neutrophil membranes	-11.3	-13.6	-17.3	67
SiO_2 NP (d: 100nm)	DMPC	-42.1	-22.4	0.082	68
SiO_2 NP (d: 40-50nm)		-39.2	-13.3		
PLA [d] NP	Platelet membrane	-31.87	-33.03	the same (N.D)	8
PLA NP-R848 [e]		-28.7	-28.2		
Escherichia coli Nissle 1917	DOPA	-38.4	-28.1	the same (N.D)	9
S. aureus		-32.4	-19.6		
E. faecalis		-28.8	-17.9		

[a] NP denotes nanoparticle; [b] NR denotes nanorod; [c] PCL-PEG denotes polycaprolactone-poly(ethylene glycol) block copolymer; [d] PLA denotes polylactic acid nanoparticle; [e] R848 represents resiquimod; N.D. denotes 'not defined'.

Supplementary Table 7. Qualitative relationships between changes in nanomaterial surface charge and the parameters of the resulting protein corona.

	Parameters of the protein corona			
	Density/ thickness	Identity/ quantity	Conformational change	Affinity
↑ Surface Charge density	Increase	No change	Increase	Increase

Supplementary Table 8. The summary of circulation time of liposomes.

**Liposome**	**$t_{1/2}$ circulation clearance lifetime (h)**	**Ref.**
EPC/CHOL/PEG-PE	5	69
PEG-SPC/CHOL	8.55	70
HSPC/CHOL/DSPE-mPEG	7.7	71
HSPC/Chol/PEG	2.17, 7.3, 7.87, 10.04	72
HSPC/CHOL/DSPE-mPEG	12.8	73
DOPE/DSPE-PEG	2.67	74
**DOPC/DSPE-PEG$_{2000}$**	**6.2**	**This work**

Supplementary Table 9. The circulation reports of empty liposome and lipid bilayer-coated nanoparticles in one work.

Nanoparticle	Circulation clearance lifetime (h)	Ref.
Liposome (DSPC/Chol/DSPE-PEG_{2000})	8.7	23
Lipid-coated mesoporous silica nanoparticle	11	
Liposome (DSPC/Chol/DSPE-PEG_{2000})	~3.3	24

Supplementary Table 10. Summary of elasticity region of the particles chose to study the relationship of elasticity and blood circulation in literature.

Elasticity	Nanoparticle	Ref.
Region I	discoidal polymeric nanoconstructs	40
Region III	zwitterionic nanogels	39
	silica nanocapsules	42
Regions I and II	hydrogel microparticles	41
Regions I and III	PEG-based hydrogel nanoparticles	38

Supplementary Table 11. The previous works in which ApoA1 proteins appeared in the protein corona of particles.

Nanoparticle	Ranking of ApoA1	Roles of ApoA1 in protein corona	Ref.
Silica nanoparticles	1	Decrease cellular uptake, relieve cytotoxicity and proinflammatory effect	75
PEGylated graphene oxide coated gold nanorods	1	Decrease macrophage uptake and prolong circulation lifetime	76
Polystyrene nanoparticles	N.D.	Decrease cellular uptake	77
Graphene nanoflakes	N.D.	Mediate cellular uptake through recognition of SR-B1 receptor	78
**75-700kPa@lipid**	**1**	**Function as a dysopsonic protein and prolong circulation lifetime**	***This work***

*N.D. denotes 'not defined'.

Supplementary Table 12. Summary on the sizes (d) of nanoparticles filtered by major organs.

Organ	Clearance	Accumulation	Ref.
Renal clearance	d: < ~5 nm		79
Hepatic clearance	d: 10-20 nm	d: 100-200 nm	80, 81
Splenic filtration		d: >200 nm	82, 83
Capillaries of lung		d: 2-5 μm	84

Supplementary Table 13. Nanoparticles covered in Region II of Figure 2c.

Nanoparticle	Composition	Ref.
Red Blood Cell Mimics Hydrogel (5.2–5.9 µm in diameter and 1.22–1.54 µm tall)	HEA [a]/PEGDA [b]	41
Discoidal Polymeric Nanoconstructs (~1000 nm in diameter and ~400 nm tall)	PLGA [c]/PEGDA/lipid-DOTA	40
liposomes	HSPC/Chol (3:1)<br>HSPC/Chol/DSPE-mPEG-2000 (3:1:1)	73
liposomes	PC/Chol/PEG-PE (1:1:0.16)	69
liposomes	SPC/Chol/PEGylated UA [d] (50:8:5)	70
liposomes	DOPE/CHEMS [e] (1.5:1)	74
liposomes	HSPC/Chol/DSPE-PEG-2000 (57:38:5)	71
liposomes	HSPC/Chol/PEG	72

[a] HEA denotes 2-hydroxyethyl acrylate; [b] PEGDA denotes poly(ethylene glycol) diacrylate; [c] PLGA denotes poly(D,L-lactide-co-glycolide); [d] UA denotes ursolic acid; [e] CHEMS denotes cholesteryl hemisuccinate.

Supplementary Figures

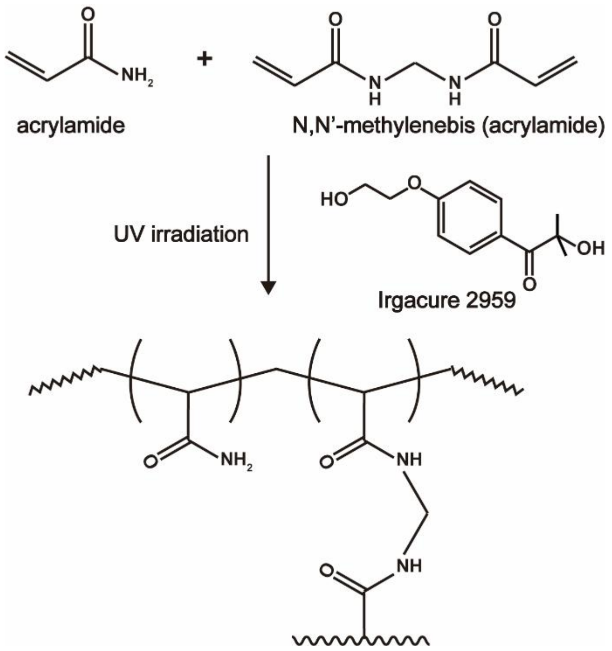

Supplementary Figure 1. Schematic illustration on the photo-polymerization of acrylamide (AA, the monomer) and N,N'-methylenebis (acrylamide) (MBA, the crosslinker) due to activation of Irgacure 2959 (the photo-initiator) upon irradiation with ultraviolet (UV) light.

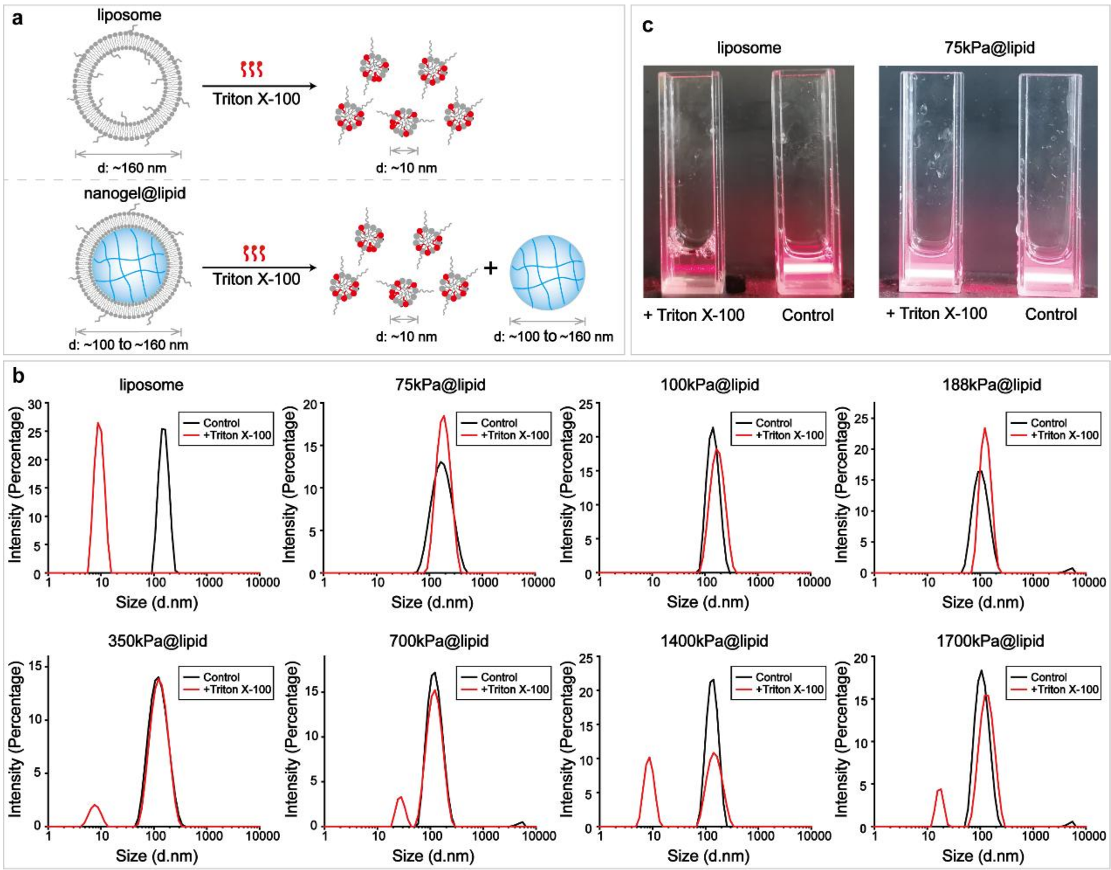

Supplementary Figure 2. **a** Schematic illustration on the distinction in changes brought by Triton X-100 treatment to liposome *versus* nanogel@lipid particles. **b** The distributions of hydrodynamic diameter by number of particles for our liposome and nanogel@lipid particles before (black) and after (red) treatment with Triton X-100. **c** Photographs of the flashlight-illuminated dispersions of liposome and 75kPa@lipid before and after treatment with Triton X-100.

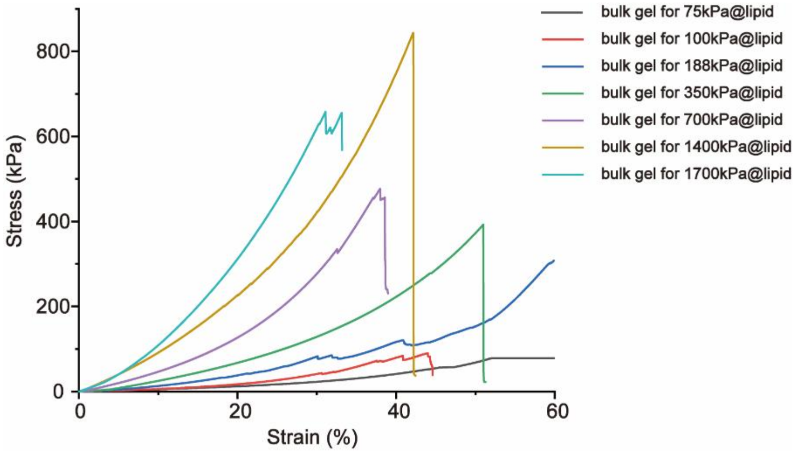

Supplementary Figure 3. The relationship between compressional force *versus* the

observed deformation of the bulk gels which were prepared with corresponding hydrogel precursor solutions used for preparing our nanogel@lipid particles.

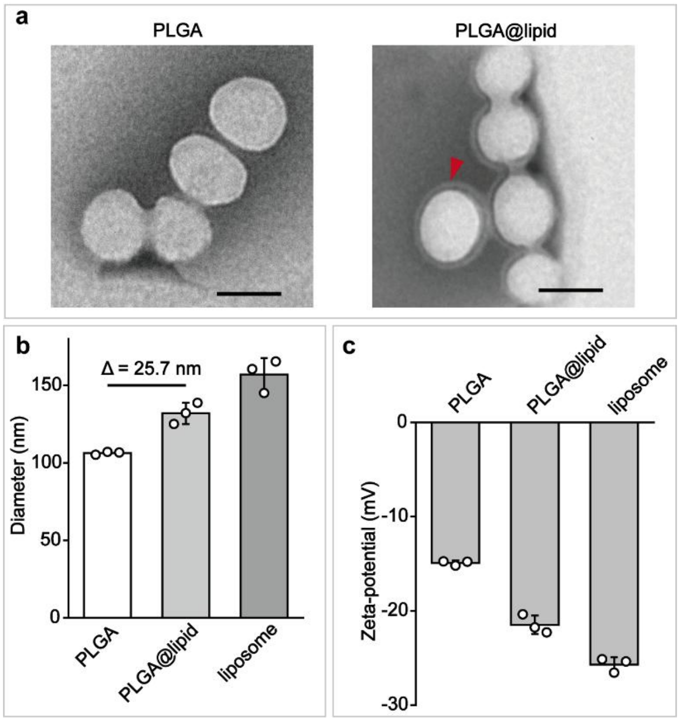

Supplementary Figure 4. Characterizations on PLGA@lipid. a TEM images of PLGA nanoparticle (left) before and (right) after coating with a lipid bilayer. For each sample, microscopy images were taken in ≥5 different microscopy fields of view, and consistent results were observed. Scale bar = 100 nm. **b** The average hydrodynamic diameter and **c** average zeta-potential of PLGA@lipid, with those of bare PLGA nanoparticle and liposome included as references. Bar heights are reported as mean ± standard deviation (n = 3 independent experiments).

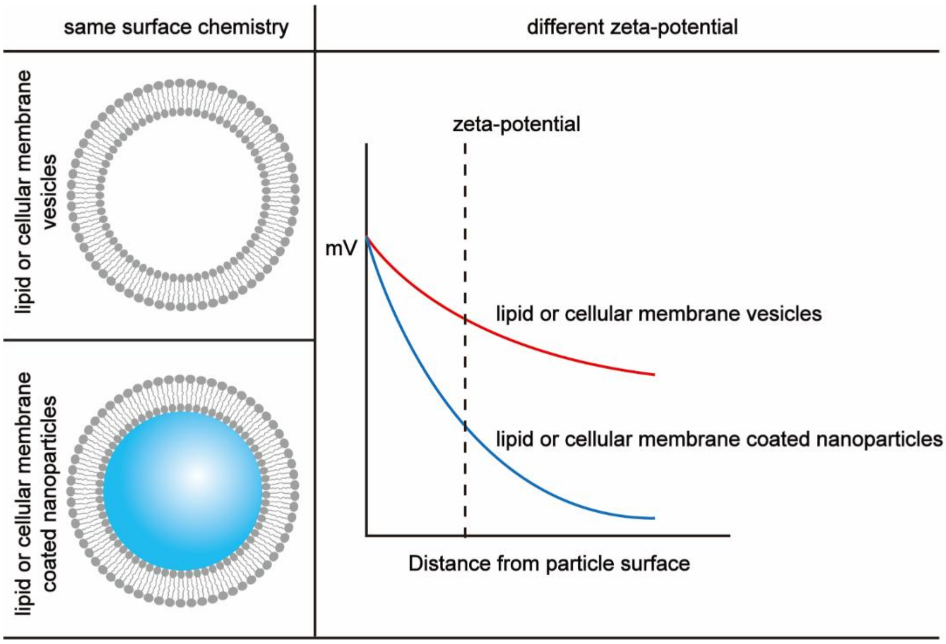

Supplementary Figure 5. The surface chemistry of both lipid or cellular membrane vesicles and lipid or cellular membrane coated nanoparticles are same, but they are different in zeta-potential due to the contribution of the particle core to partially screen the charges on the outermost layer of the particle.

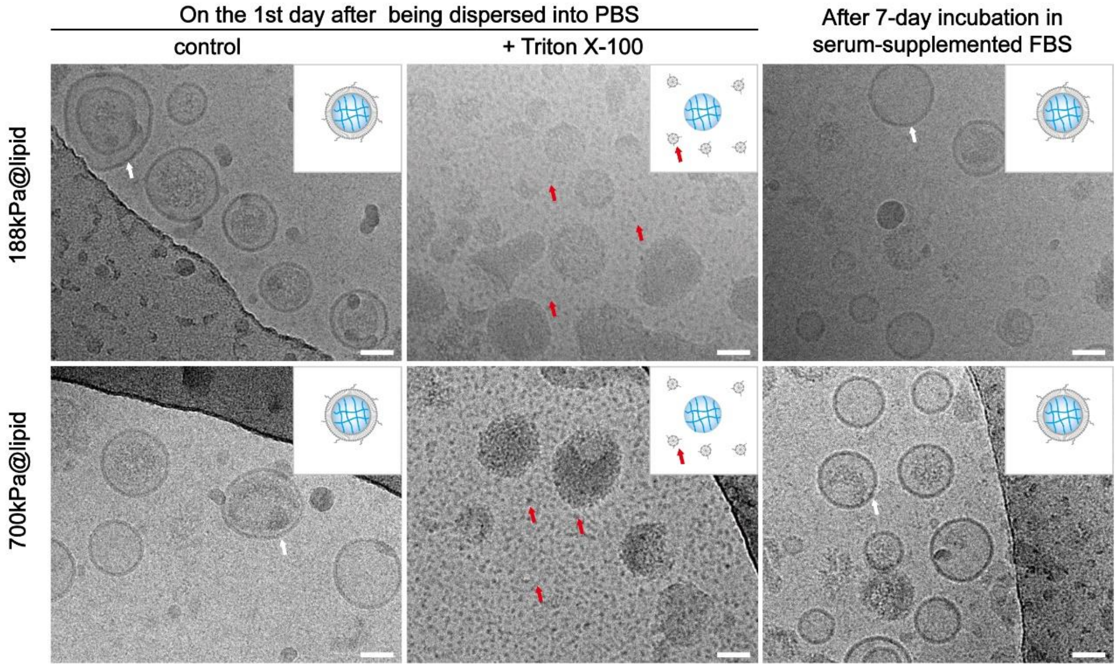

Supplementary Figure 6. Cryo-electron microscope (Cryo-EM) images of our (top) 188kPa@lipid and (bottom) 700kPa@lipid nanoparticles (left) on the 1st day after being dispersed into PBS, (middle) after treatment with Triton X-100, and (right) after

7-day incubation in fetal bovine serum (FBS)-supplemented phosphate buffered saline PBS (v./v., 50%). White arrows indicate the locations of the lipid bilayers, and red arrows indicate small micellar particles composed of lipids peeled off our core-shell structured nanoparticles. (Inset) Schematic illustrations on nanoparticles respectively present in the corresponding dispersion samples. For each sample, microscopy images were taken in ≥5 different microscopy fields of view, and consistent results were observed. Scale bar = 50 nm.

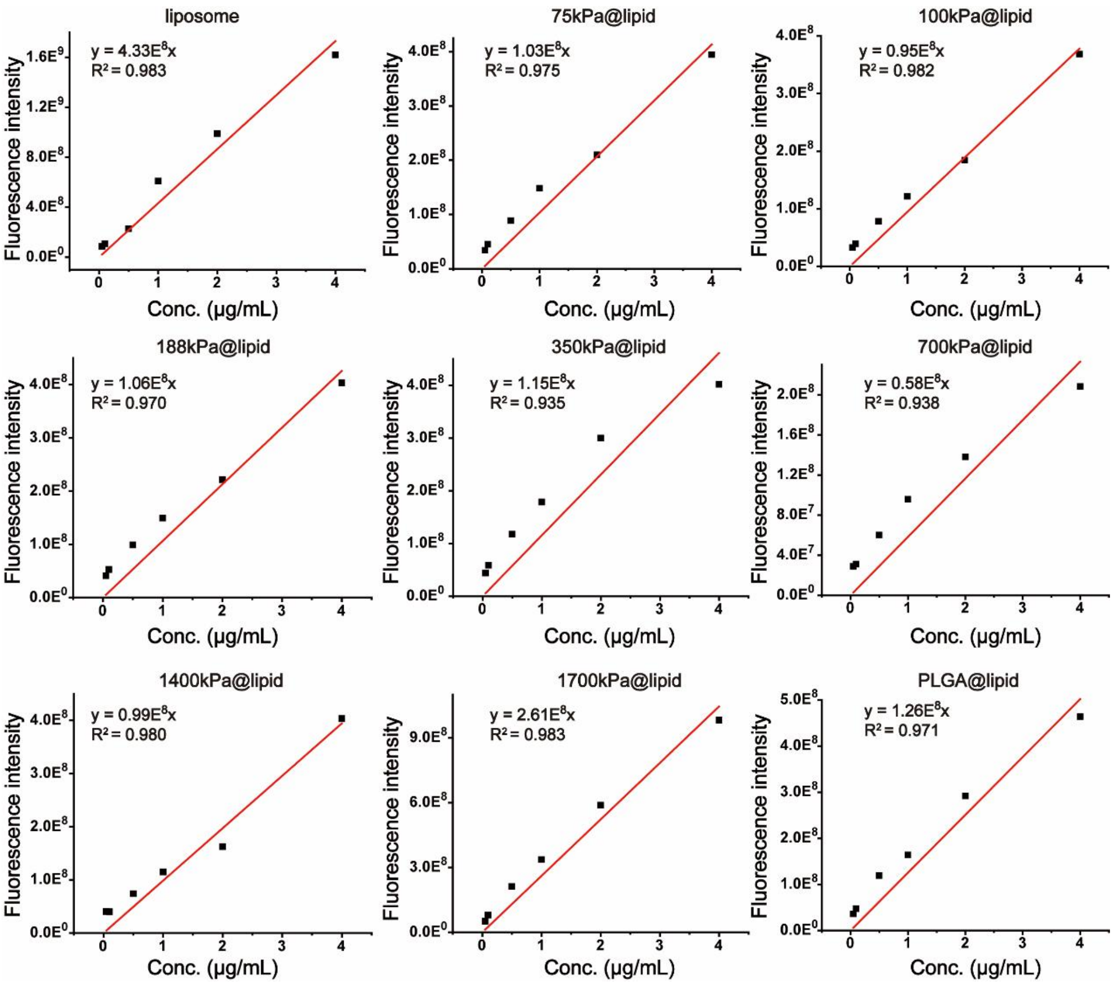

Supplementary Figure 7. Calibration curves on the relationship between a nanoparticle's fluorescence intensity at 680 nm *versus* DiD concentration for our DiD-labeled nanoparticles.

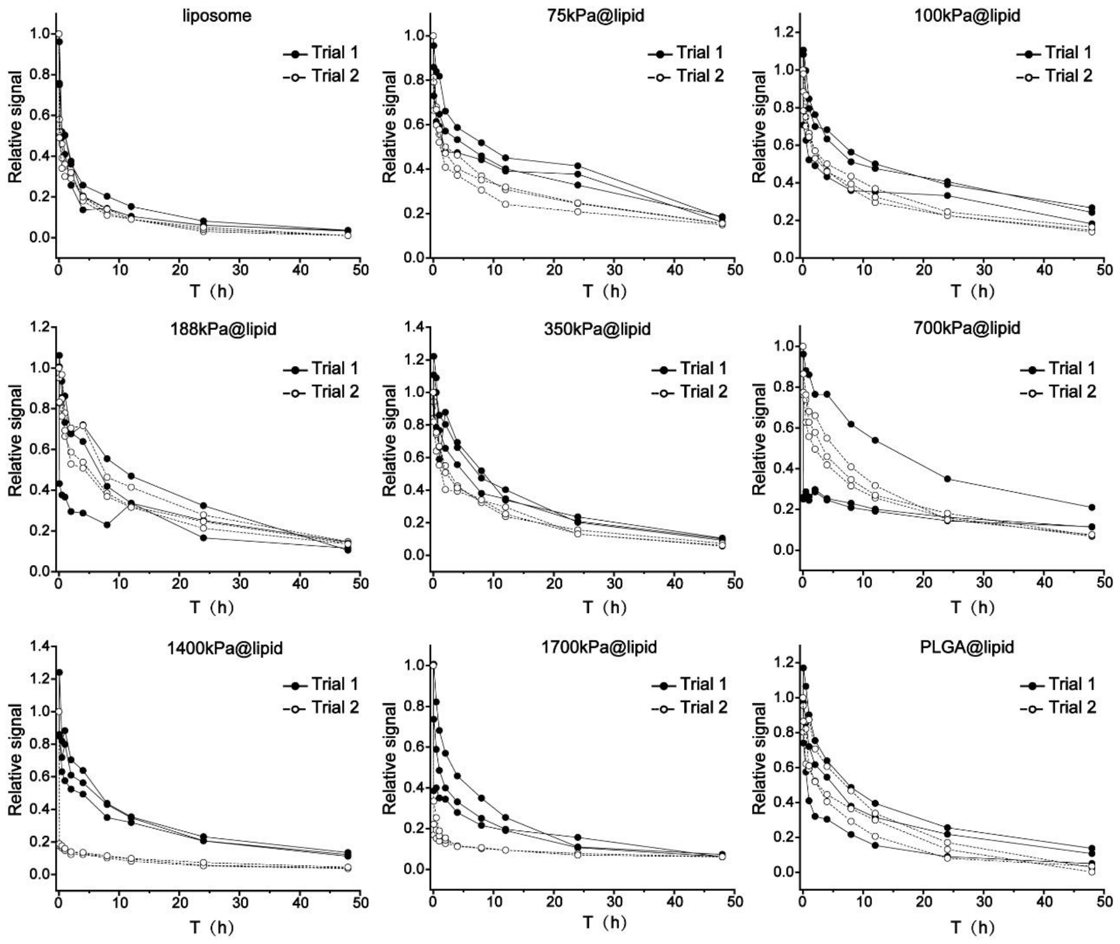

Supplementary Figure 8. Blood retention profiles of our PEGylated model nanoparticles (DiD-labeled) in healthy ICR (CD-1) mice from two independent trials (n = 3 biologically independent mice in each independent trial).

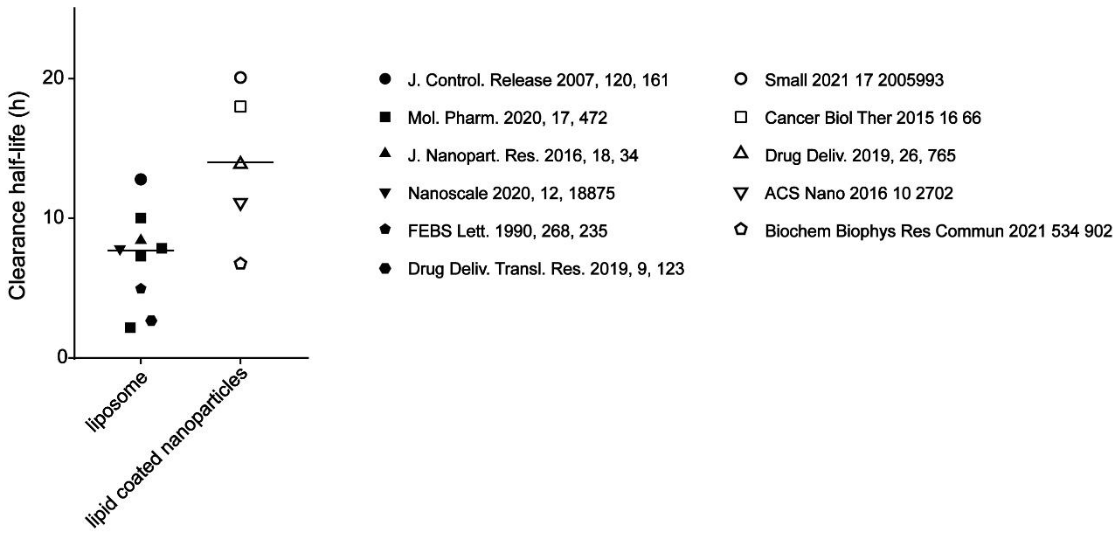

Supplementary Figure 9. The clearance half-lives of liposome and lipid coated stiff nanoparticles in literatures.

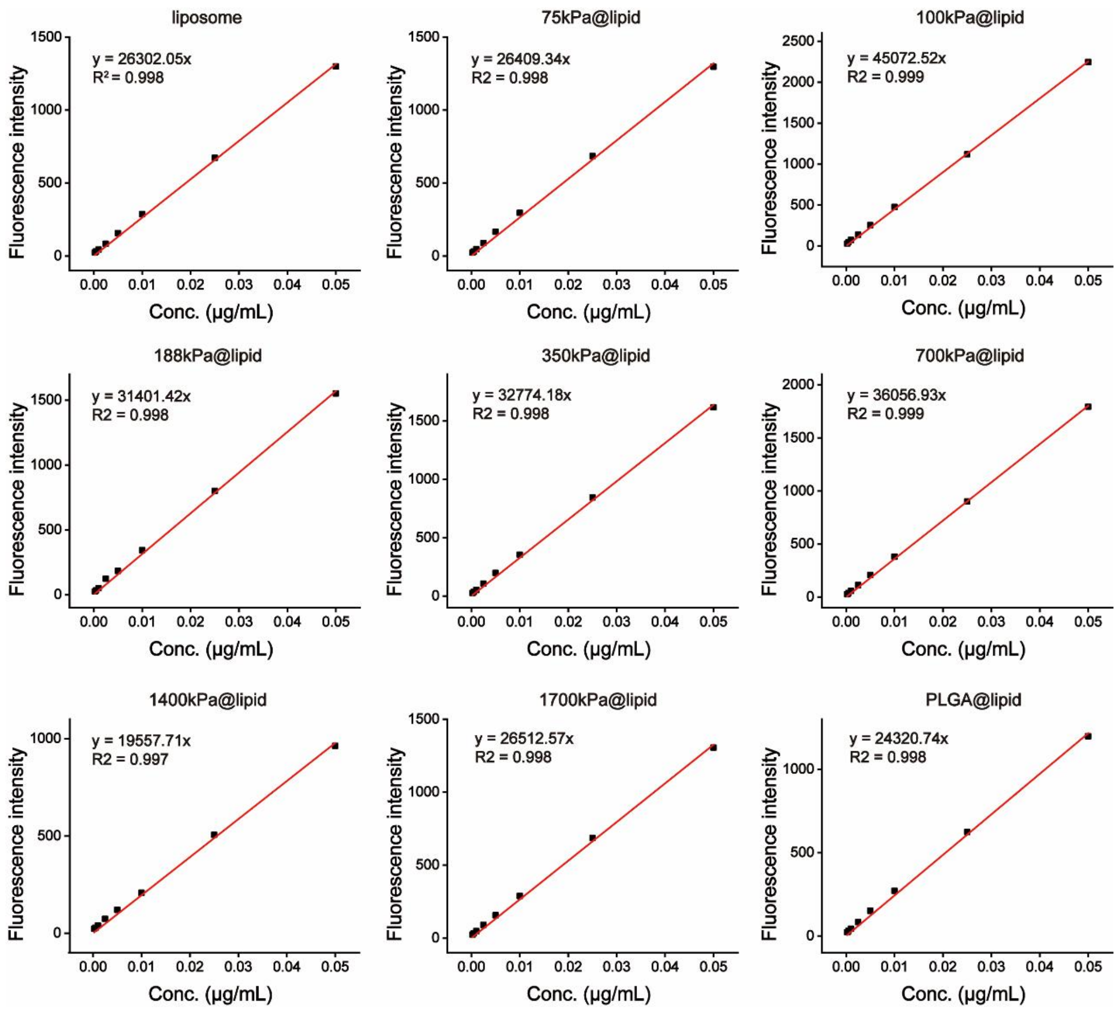

Supplementary Figure 10. Calibration curves on the relationship between a nanoparticle's fluorescence intensity at 569 nm *versus* DiI concentration for our

Dil-labeled nanoparticles.

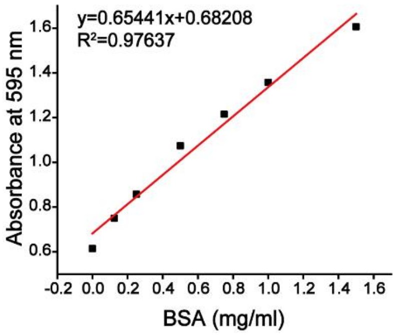

Supplementary Figure 11. Calibration curve on the relationship between nanoparticle's absorbance at 595 nm *versus* concentration of bovine serum albumin (BSA), used quantifying protein amounts with a Bradford kit.

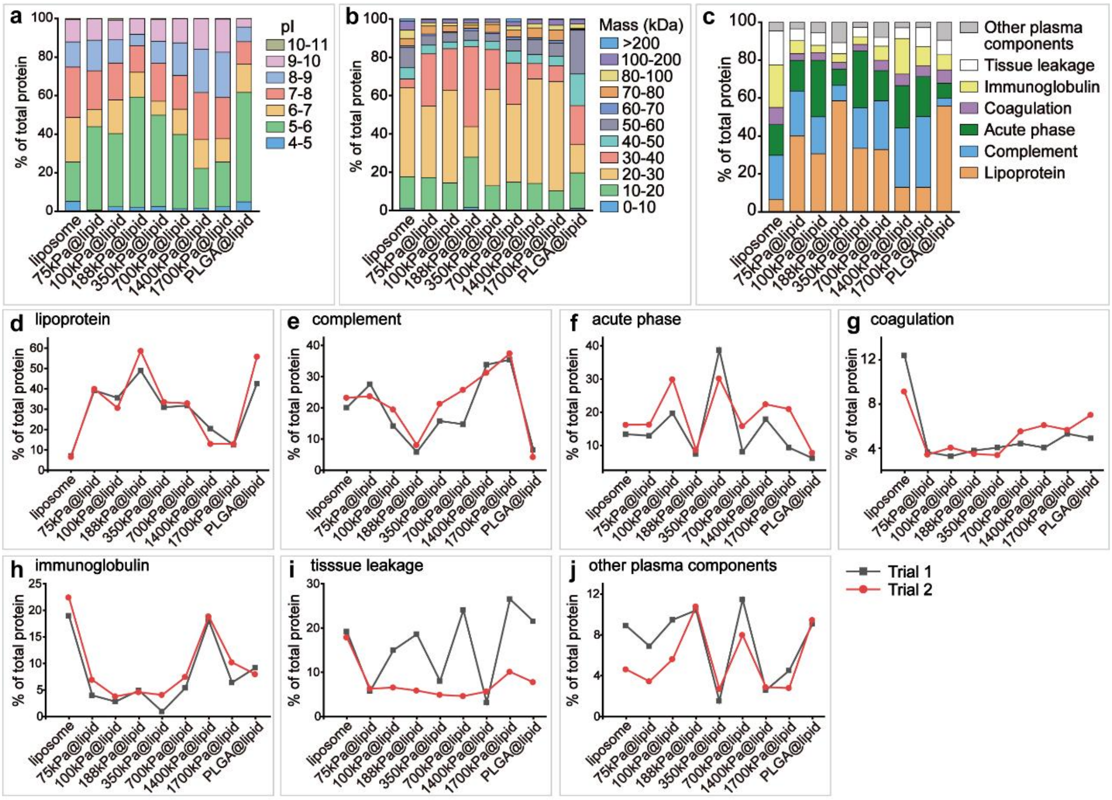

Supplementary Figure 12. Results from trial 2 on protein corona over our PEGylated model nanoparticles. a-c Classification of corona proteins according to **(a)** molecular weight, **(b)** calculated isoelectric point (pI), and **(c)** physiological functions. **d-j** Comparison on the distribution of the relative abundance in corona of trial 1 and trial 2 for our PEGylated nanoparticles.

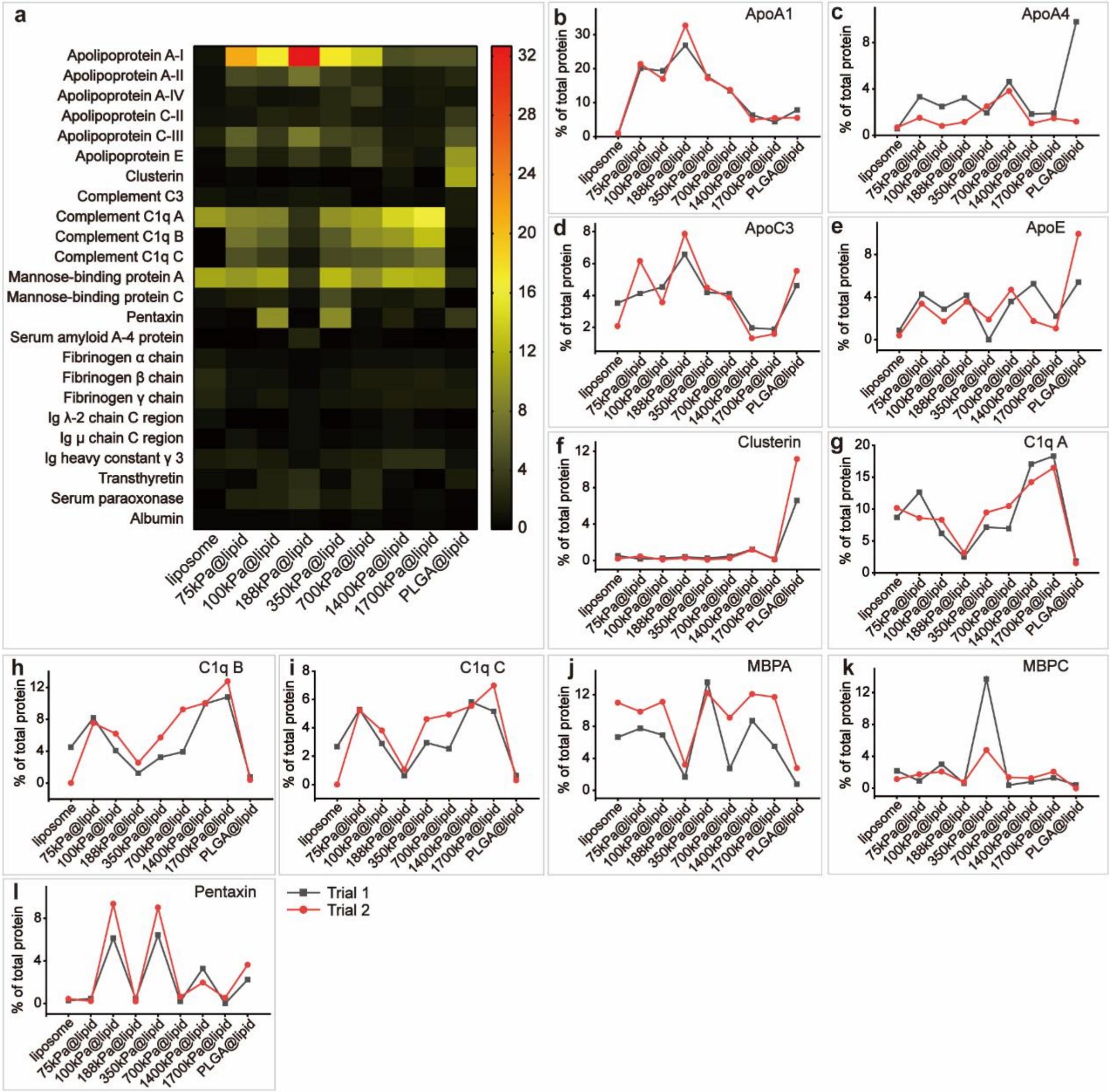

Supplementary Figure 13. Results from trial 2 on protein corona over our PEGylated model nanoparticles. a Heat map of the most abundant proteins in protein coronas of our nanoparticles. **b-l** Comparison on the distribution of the relative abundance in corona of trial 1 and trial 2 for our PEGylated nanoparticles for a protein which exhibited a relative abundance of >5% on at least one nanoparticle.

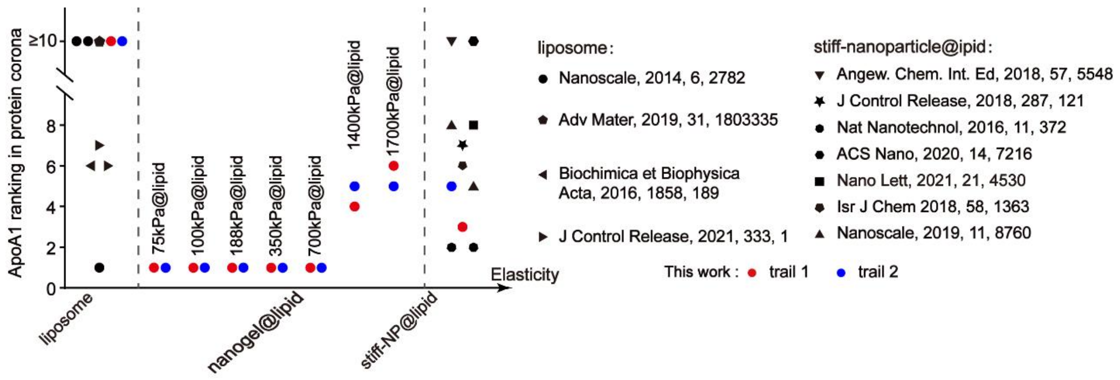

Supplementary Figure 14. Comparison on the ranking of ApoA1 in protein coronas of our nanoparticles as well as in those of PEGylated liposomes and PEGylated lipid bilayer-coated stiff-nanoparticles in prior reports.

Trail 1

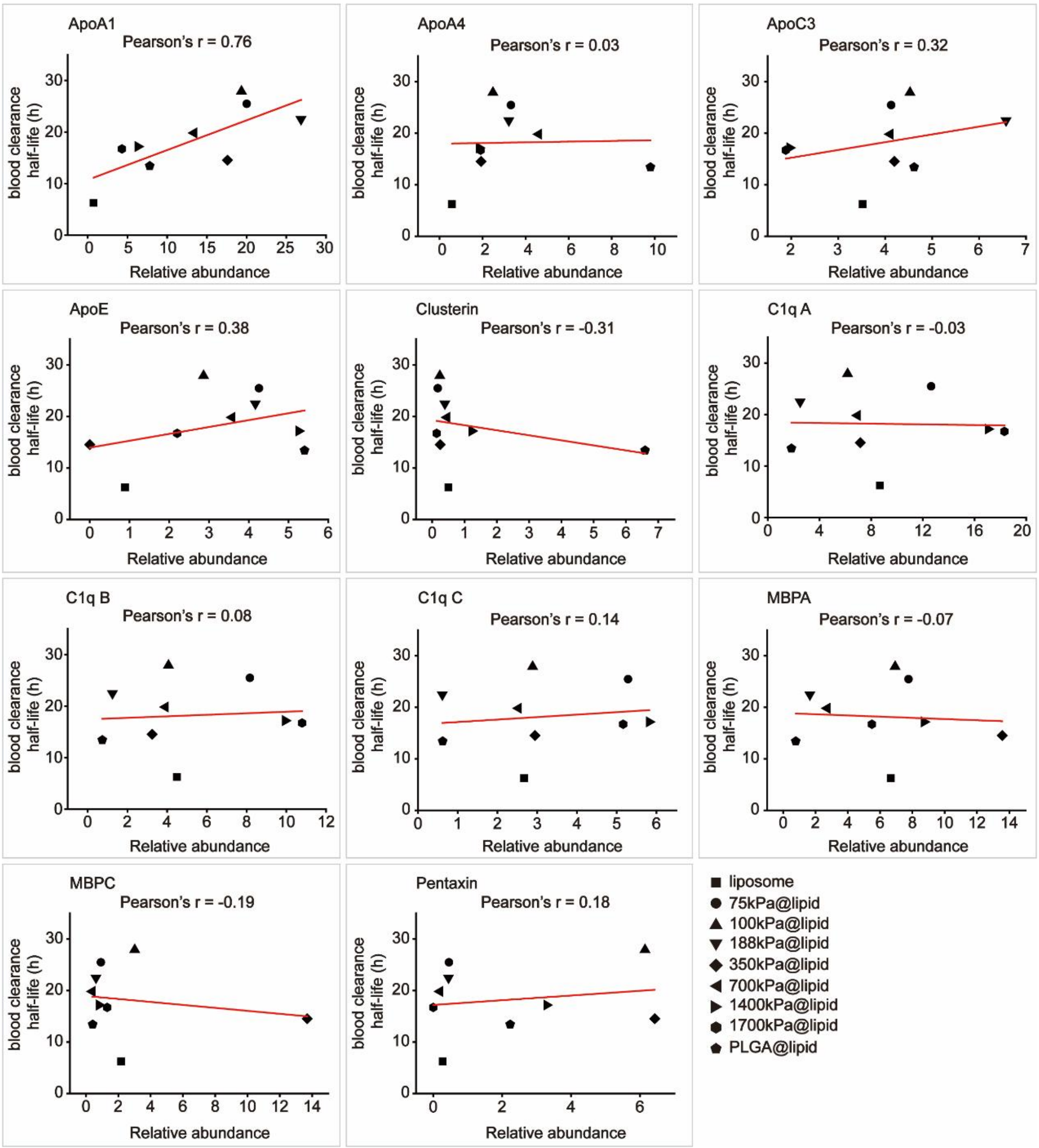

Supplementary Figure 15. Plots on the relationship between the relative abundance in corona of PEGylated lipid coated nanoparticles *versus* their blood clearance half-lives , from which Pearson's correlation coefficient r between relative abundance in corona and nanoparticle blood clearance half-life was calculated for a specific protein with a relative abundance >5% on any particle. Data on protein corona content are from trial 1.

Trial 2

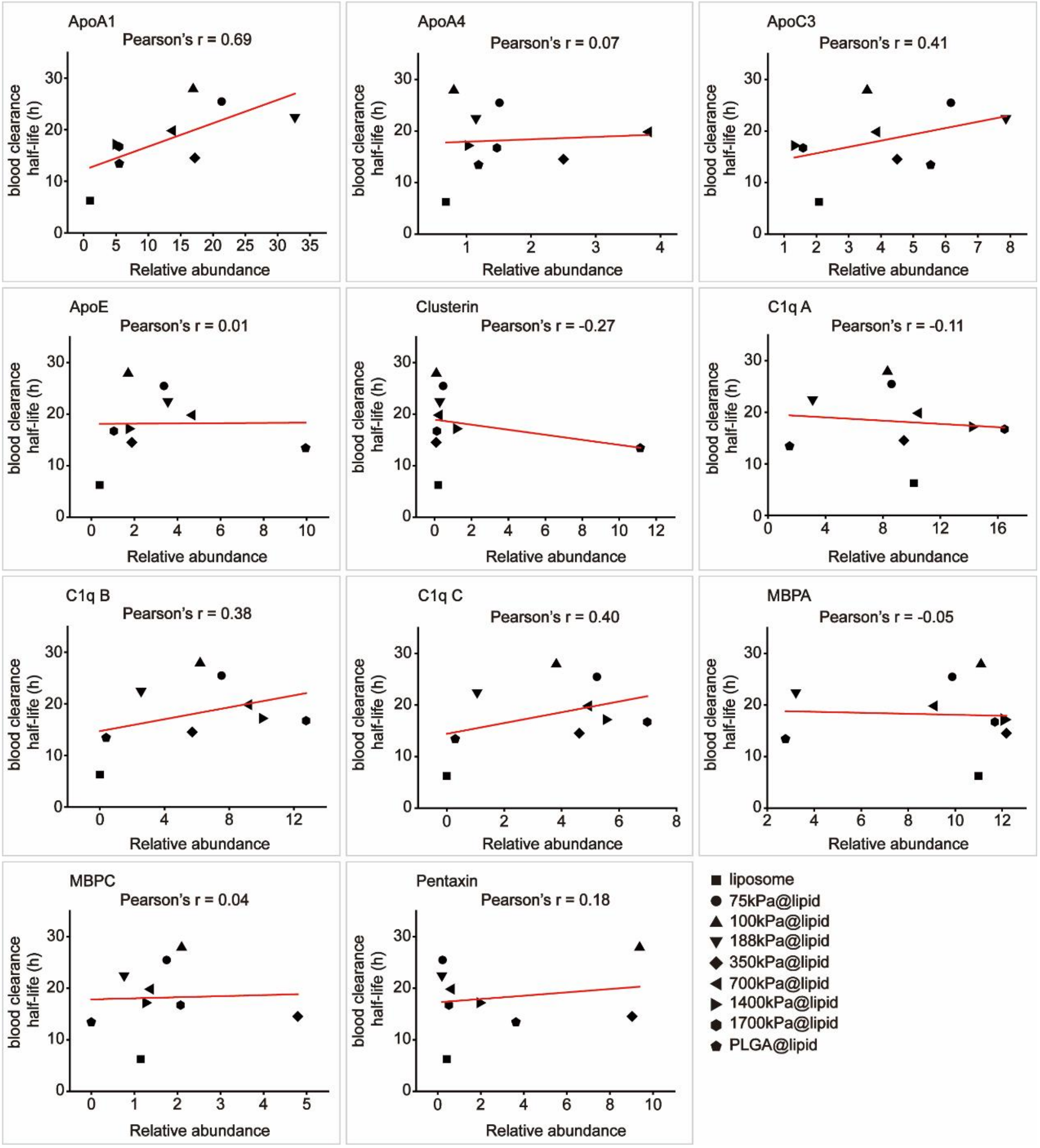

Supplementary Figure 16. Plots on the relationship between the relative abundance in corona of PEGylated lipid coated nanoparticles *versus* their blood clearance half-lives, from which Pearson's correlation coefficient r between relative abundance in corona and nanoparticle blood clearance half-life was calculated for a specific protein with a relative abundance >5% on any particle. Data on protein corona content are from trial 2.

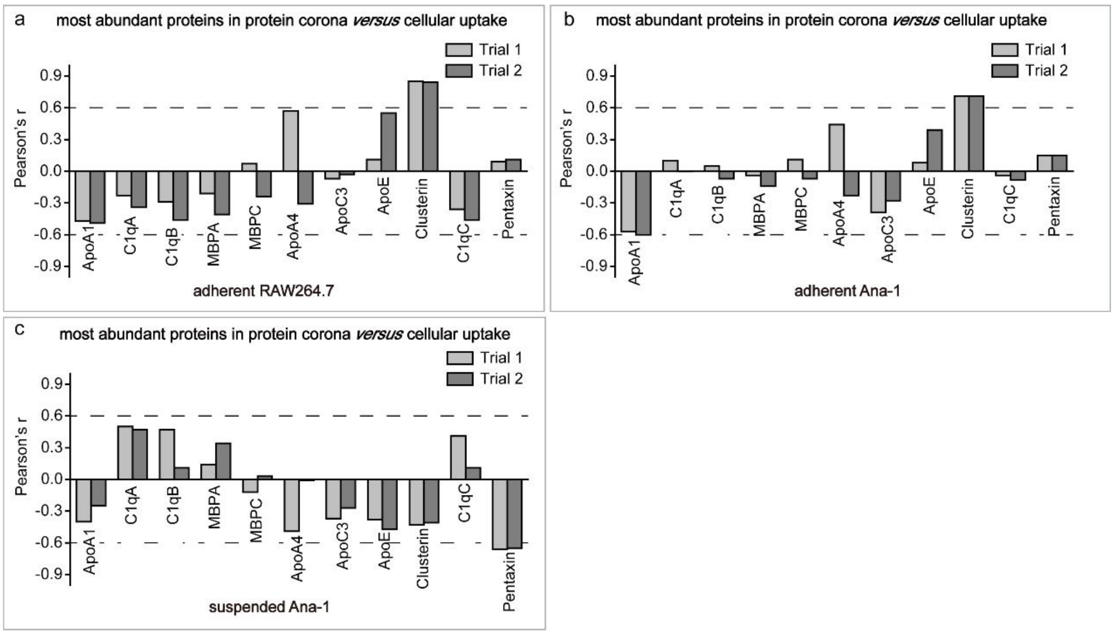

Supplementary Figure 17. Pearson's r between the relative abundance in corona and nanoparticle cellular uptake of **a** adherent RAW264.7, **b** adherent Ana-1 and **c** suspended Ana-1 for proteins which exhibited a relative abundance of >5% on at least one nanoparticle.

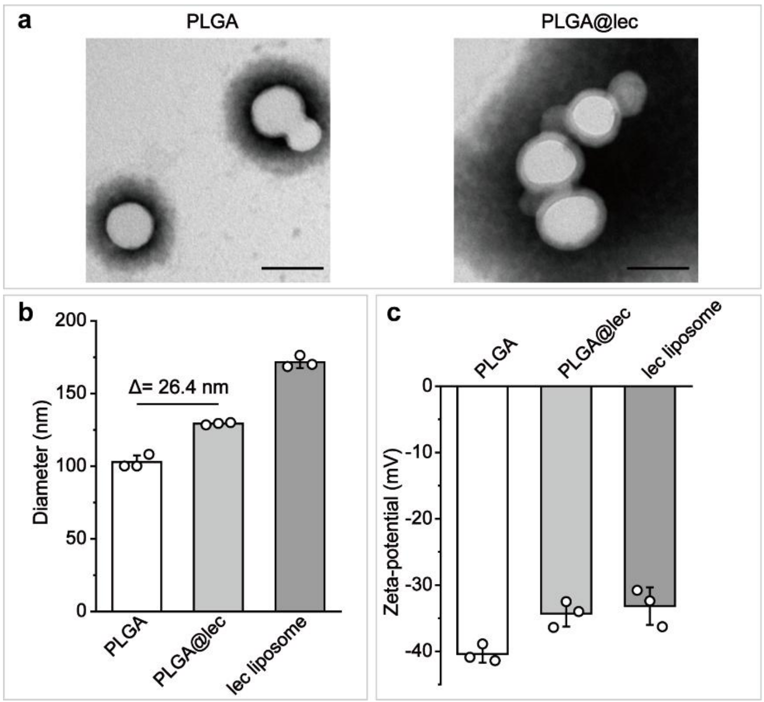

Supplementary Figure 18. Characterizations on PLGA@lec. a TEM images of PLGA nanoparticles (left) before and (right) after coating with a lethicin (lec) bilayer.

For each sample, microscopy images were taken in ≥5 different microscopy fields of view, and consistent results were observed. Scale bar = 100 nm. **b** The average hydrodynamic diameter and **c** average zeta-potential of PLGA@lec nanoparticle, with those of the lec liposome and bare PLGA nanoparticle included as references. Bar heights are reported as mean ± standard deviation (n = 3 independent experiments).

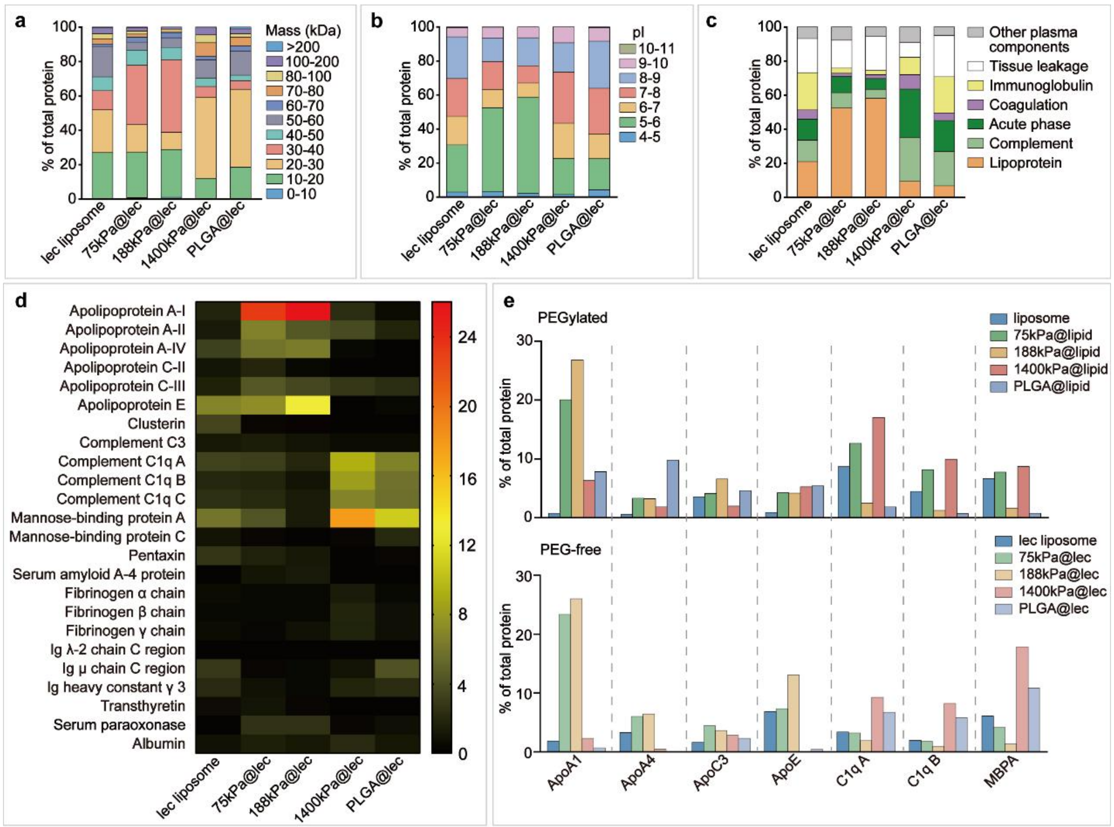

Supplementary Figure 19. Results from trial 2 on protein coronas over our PEG-free model nanoparticles. a-c Classifications of corona proteins according to **(a)** molecular weight, **(b)** calculated isoelectric point (pI), and **(c)** physiological functions for our PEG-free nanoparticles. **d** Heat map of the most abundant proteins in the coronas on our PEG-free nanoparticles. **e** Comparison on the distribution of the relative abundance in coronas on our (top) PEGylated and (bottom) PEG-free nanoparticles for proteins which exhibited a relative abundance in corona of >5% on at least one nanoparticle.

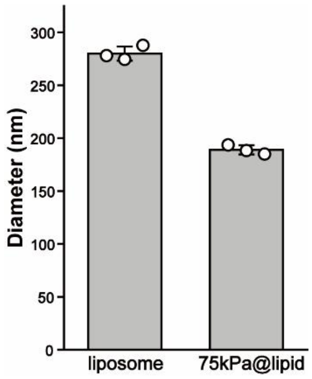

Supplementary Figure 20. The average hydrodynamic diameter of 75kPa@lipid nanoparticle prepared by extruding hydrogel precursor solution-preloaded liposome (DOPC : DSPE-PEG_{2000} = 90 : 10) through polycarbon nucleopore membrane with 400-nm pores followed by photo-polymerization. That of liposome (DOPC : DSPE-PEG_{2000} = 90 : 10) prepared by extruding through polycarbon nucleopore membrane with 400-nm pores was included for comparison. Bar heights are reported as mean ± standard deviation (n = 3 independent experiments).

SUPPLEMENTARY REFERENCES

1. Kazakov, S., Kaholek, M., Teraoka, I. & Levon, K. UV-Induced Gelation on Nanometer Scale Using Liposome Reactor. Macromolecules 35, 1911-1920 (2002).
2. Gao, J. et al. PEGylated lipid bilayer coated mesoporous silica nanoparticles co-delivery of paclitaxel and curcumin leads to increased tumor site drug accumulation and reduced tumor burden. Eur. J. Pharm. Sci. 140, 105070 (2019).
3. Li, Z. et al. Folic acid modified lipid-bilayer coated mesoporous silica nanoparticles co-loading paclitaxel and tanshinone IIA for the treatment of acute promyelocytic leukemia. Int. J. Pharm. 586, 119576 (2020).
4. Lin, J. et al. PEGylated Lipid bilayer coated mesoporous silica nanoparticles for co-delivery of paclitaxel and curcumin: Design, characterization and its cytotoxic effect. Int. J. Pharm. 536, 272-282 (2018).
5. Wang, Z. & Ho, P.C. Self-Assembled Core–Shell Vascular-Targeted Nanocapsules for Temporal Antivasculature and Anticancer Activities. Small 6, 2576-2583 (2010).
6. Ahmaditabar, P. et al. Enhanced Entrapment and Improved in Vitro Controlled Release of N-Acetyl Cysteine in Hybrid PLGA/Lecithin Nanoparticles Prepared Using a Nanoprecipitation/Self-Assembly Method. J. Cell. Biochem. 118, 4203-4209 (2017).
7. Sedki, M., Khalil, I.A. & El-Sherbiny, I.M. Hybrid nanocarrier system for guiding and augmenting simvastatin cytotoxic activity against prostate cancer. Artif Cells Nanomed Biotechnol 46, S641-S650 (2018).
8. Bahmani, B. et al. Intratumoral immunotherapy using platelet-cloaked nanoparticles enhances antitumor immunity in solid tumors. Nat. Commun. 12, 1999 (2021).
9. Cao, Z., Wang, X., Pang, Y., Cheng, S. & Liu, J. Biointerfacial self-assembly generates lipid membrane coated bacteria for enhanced oral delivery and treatment. Nat. Commun. 10, 5783 (2019).
10. Savarala, S., Ahmed, S., Ilies, M.A. & Wunder, S.L. Formation and Colloidal

Stability of DMPC Supported Lipid Bilayers on SiO2 Nanobeads. Langmuir 26, 12081-12088 (2010).
11. Yusko, E.C. et al. Controlling protein translocation through nanopores with bio-inspired fluid walls. Nat. Nanotechnol. 6, 253-260 (2011).
12. Sieber, S. et al. Zebrafish as a predictive screening model to assess macrophage clearance of liposomes in vivo. Nanomedicine 17, 82-93 (2019).
13. Ishida, T. et al. Accelerated blood clearance of PEGylated liposomes following preceding liposome injection: Effects of lipid dose and PEG surface-density and chain length of the first-dose liposomes. J. Control. Release 105, 305-317 (2005).
14. Song, Y. et al. Are third-generation active-targeting nanoformulations definitely the best? In vitro and in vivo comparisons of pixantrone-loaded liposomes modified with different sialic acid derivatives. Drug Deliv. Transl. Res. 12, 647-661 (2022).
15. Troutier, A.L. & Ladaviere, C. An overview of lipid membrane supported by colloidal particles. Adv. Colloid Interface Sci. 133, 1-21 (2007).
16. ZHANG, L. & ZHANG, L. Lipid–polymer hybrid nanoparticles: synthesis, characterization and applications. Nano LIFE 1, 163-173 (2010).
17. Obringer, A.R., Rote, N.S. & Walter, A. Antiphospholipid antibody binding to bilayer-coated glass microspheres. J. Immunol. Methods 185, 81-93 (1995).
18. Buranda, T. et al. Biomimetic molecular assemblies on glass and mesoporous silica microbeads for biotechnology. Langmuir 19, 1654-1663 (2003).
19. Gessner, A., Lieske, A., Paulke, B.R. & Müller, R.H. Influence of surface charge density on protein adsorption on polymeric nanoparticles: analysis by two-dimensional electrophoresis. Eur. J. Pharm. Biopharm. 54, 165-170 (2002).
20. Walkey, C.D. & Chan, W.C.W. Understanding and controlling the interaction of nanomaterials with proteins in a physiological environment. Chem. Soc. Rev. 41, 2780-2799 (2012).
21. Lundqvist, M. et al. Nanoparticle size and surface properties determine the protein corona with possible implications for biological impacts. Proc. Natl. Acad. Sci. U. S. A. 105, 14265-14270 (2008).
22. Gessner, A., Lieske, A., Paulke, B.R. & Muller, R.H. Functional groups on

polystyrene model nanoparticles: Influence on protein adsorption. J. Biomed. Mater. Res. Part A 65A, 319-326 (2003).

23. Liu, X.S. et al. Irinotecan Delivery by Lipid-Coated Mesoporous Silica Nanoparticles Shows Improved Efficacy and Safety over Liposomes for Pancreatic Cancer. ACS Nano 10, 2702-2715 (2016).

24. Liu, X.S. et al. Improved Efficacy and Reduced Toxicity Using a Custom-Designed Irinotecan-Delivering Silicasome for Orthotopic Colon Cancer. ACS Nano 13, 38-53 (2019).

25. Clemments, A.M., Botella, P. & Landry, C.C. Protein Adsorption From Biofluids on Silica Nanoparticles: Corona Analysis as a Function of Particle Diameter and Porosity. ACS Appl. Mater. Interfaces 7, 21682-21689 (2015).

26. Hadjidemetriou, M. et al. The Human In Vivo Biomolecule Corona onto PEGylated Liposomes: A Proof-of-Concept Clinical Study. Adv. Mater. 31, 1803335 (2019).

27. Tenzer, S. et al. Nanoparticle Size Is a Critical Physicochemical Determinant of the Human Blood Plasma Corona: A Comprehensive Quantitative Proteomic Analysis. ACS Nano 5, 7155-7167 (2011).

28. Aggarwal, P., Hall, J.B., McLeland, C.B., Dobrovolskaia, M.A. & McNeil, S.E. Nanoparticle interaction with plasma proteins as it relates to particle biodistribution, biocompatibility and therapeutic efficacy. Adv. Drug Deliv. Rev. 61, 428-437 (2009).

29. Nel, A.E. et al. Understanding biophysicochemical interactions at the nano–bio interface. Nat. Mater. 8, 543-557 (2009).

30. Dobrovolskaia, M.A. et al. Interaction of colloidal gold nanoparticles with human blood: effects on particle size and analysis of plasma protein binding profiles. Nanomedicine 5, 106-117 (2009).

31. Mahmoudi, M. et al. Protein−Nanoparticle Interactions: Opportunities and Challenges. Chem. Rev. 111, 5610-5637 (2011).

32. Cedervall, T. et al. Understanding the nanoparticle–protein corona using methods to quantify exchange rates and affinities of proteins for nanoparticles. Proc. Natl.

Acad. Sci. U. S. A. 104, 2050-2055 (2007).
33. Lynch, I. & Dawson, K.A. Protein-nanoparticle interactions. Nano Today 3, 40-47 (2008).
34. Schöttler, S. et al. Protein adsorption is required for stealth effect of poly(ethylene glycol)- and poly(phosphoester)-coated nanocarriers. Nat. Nanotechnol. 11, 372-377 (2016).
35. Puppione, D.L. et al. Mass spectral analysis of the apolipoproteins on mouse high density lipoproteins. Detection of post-translational modifications. Biochim Biophys Acta Proteins Proteom 1764, 1363-1371 (2006).
36. Pankhurst, G. et al. Characterization of specifically oxidized apolipoproteins in mildly oxidized high density lipoprotein. J. Lipid Res. 44, 349-355 (2003).
37. Reschly, E.J. et al. Apolipoprotein A-I α-Helices 7 and 8 Modulate High Density Lipoprotein Subclass Distribution*. J. Biol. Chem. 277, 9645-9654 (2002).
38. Anselmo, A.C. et al. Elasticity of Nanoparticles Influences Their Blood Circulation, Phagocytosis, Endocytosis, and Targeting. ACS Nano 9, 3169-3177 (2015).
39. Zhang, L. et al. Softer Zwitterionic Nanogels for Longer Circulation and Lower Splenic Accumulation. ACS Nano 6, 6681-6686 (2012).
40. Key, J. et al. Soft Discoidal Polymeric Nanoconstructs Resist Macrophage Uptake and Enhance Vascular Targeting in Tumors. ACS Nano 9, 11628-11641 (2015).
41. Merkel, T.J. et al. Using mechanobiological mimicry of red blood cells to extend circulation times of hydrogel microparticles. Proc. Natl. Acad. Sci. U. S. A. 108, 586-591 (2011).
42. Hui, Y. et al. Understanding the Effects of Nanocapsular Mechanical Property on Passive and Active Tumor Targeting. ACS Nano 12, 2846-2857 (2018).
43. Beningo, K.A. & Wang, Y.-l. Fc-receptor-mediated phagocytosis is regulated by mechanical properties of the target. J. Cell. Sci. 115, 849-856 (2002).
44. Alexander, J.F. et al. Cubical Shape Enhances the Interaction of Layer-by-Layer Polymeric Particles with Breast Cancer Cells. Adv. Healthc. Mater. 4, 2657-2666 (2015).

45. Teng, Z. et al. Deformable Hollow Periodic Mesoporous Organosilica Nanocapsules for Significantly Improved Cellular Uptake. J. Am. Chem. Soc. 140, 1385-1393 (2018).
46. Banquy, X. et al. Effect of mechanical properties of hydrogel nanoparticles on macrophage cell uptake. Soft Matter 5, 3984-3991 (2009).
47. Cui, J. et al. Mechanically tunable, self-adjuvanting nanoengineered polypeptide particles. Adv. Mater. 25, 3468-3472 (2013).
48. Müllner, M. et al. Size and Rigidity of Cylindrical Polymer Brushes Dictate Long Circulating Properties In Vivo. ACS Nano 9, 1294-1304 (2015).
49. Brochu, H. & Vermette, P. Young's moduli of surface-bound liposomes by atomic force microscopy force measurements. Langmuir 24, 2009-2014 (2008).
50. Guo, P. et al. Nanoparticle elasticity directs tumor uptake. Nat. Commun. 9, 130-138 (2018).
51. Liang, X.M., Mao, G.Z. & Ng, K.Y.S. Mechanical properties and stability measurement of cholesterol-containing liposome on mica by atomic force microscopy. J. Colloid Interface Sci. 278, 53-62 (2004).
52. Et-Thakafy, O. et al. Mechanical Properties of Membranes Composed of Gel-Phase or Fluid-Phase Phospholipids Probed on Liposomes by Atomic Force Spectroscopy. Langmuir 33, 5117-5126 (2017).
53. Franze, S. et al. Tuning the Extent and Depth of Penetration of Flexible Liposomes in Human Skin. Mol. Pharm. 14, 1998-2009 (2017).
54. Benne, N. et al. Atomic force microscopy measurements of anionic liposomes reveal the effect of liposomal rigidity on antigen-specific regulatory T cell responses. J. Control. Release 318, 246-255 (2020).
55. Radmacher, M., Fritz, M., Kacher, C.M., Cleveland, J.P. & Hansma, P.K. Measuring the viscoelastic properties of human platelets with the atomic force microscope. Biophys. J. 70, 556-567 (1996).
56. Bushell, G.R. et al. Imaging and force-distance analysis of human fibroblasts in vitro by atomic force microscopy. Cytometry 36, 254-264 (1999).

57. Lee, Y.J., Patel, D. & Park, S. Local Rheology of Human Neutrophils Investigated Using Atomic Force Microscopy. Int. J. Biol. Sci. 7, 102-111 (2011).
58. Dulinska Molak, I. et al. Stiffness of normal and pathological erythrocytes studied by means of atomic force microscopy. J. Biochem. Biophys. Methods 66, 1-11 (2006).
59. Wang, J., Yu, M., Liu, L., Zhao, J. & Wang, H. Mechanical Characterization of Hepatoma Cells Using Atomic Force Microscope. Materials Science Forum 694, 869-873 (2011).
60. Lekka, M. et al. Elasticity of normal and cancerous human bladder cells studied by scanning force microscopy. Eur. Biophys. J. Biophys. Lett. 28, 312-316 (1999).
61. Leporatti, S. et al. Elasticity and adhesion of resting and lipopolysaccharide-stimulated macrophages. FEBS Lett. 580, 450-454 (2006).
62. Hu, K., Zhao, F. & Wang, Q. Mechanical characterization of living and dead undifferentiated human adipose-derived stem cells by using atomic force microscopy. Proc. Inst. Mech. Eng. [H] 227, 1319-1323 (2013).
63. Troutier, A.L., Delair, T., Pichot, C. & Ladaviere, C. Physicochemical and interfacial investigation of lipid/polymer particle assemblies. Langmuir 21, 1305-1313 (2005).
64. JH, K., YT, K. & SSA, A. Lipid-coated gold nanocomposites for enhanced cancer therapy. International Journal of Nanomedicine 2015, 33—45 (2015).
65. Ancona, A. et al. Lipid-Coated Zinc Oxide Nanoparticles as Innovative ROS-Generators for Photodynamic Therapy in Cancer Cells. Nanomaterials 8, 143 (2018).
66. Xiong, J.Q. et al. Cancer-Erythrocyte Hybrid Membrane-Camouflaged Magnetic Nanoparticles with Enhanced Photothermal-Immunotherapy for Ovarian Cancer. ACS Nano 15, 19756-19770 (2021).
67. Wang, K.Y. et al. Neutrophil membranes coated, antibiotic agent loaded nanoparticles targeting to the lung inflammation. Colloid Surf. B-Biointerfaces 188, 110755 (2020).
68. Wang, K. et al. Neutrophil membranes coated, antibiotic agent loaded

nanoparticles targeting to the lung inflammation. Colloids and Surfaces B: Biointerfaces 188, 110755 (2020).
69. Klibanov, A.L., Maruyama, K., Torchilin, V.P. & Huang, L. Amphipathic polyethyleneglycols effectively prolong the circulation time of liposomes. FEBS Lett. 268, 235-237 (1990).
70. Wang, Q. et al. An evaluation of anti-tumor effect and toxicity of PEGylated ursolic acid liposomes. J. Nanopart. Res. 18, 34 (2016).
71. Viana, I.M.d.O. et al. Role of the complement cascade in the biological fate of liposomes in rodents. Nanoscale 12, 18875-18884 (2020).
72. Mastrotto, F. et al. In Vitro and in Vivo Behavior of Liposomes Decorated with PEGs with Different Chemical Features. Mol. Pharm. 17, 472-487 (2020).
73. Han, H.D. et al. Enhanced circulation time and antitumor activity of doxorubicin by comblike polymer-incorporated liposomes. J. Control. Release 120, 161-168 (2007).
74. Nunes, S.S. et al. Influence of PEG coating on the biodistribution and tumor accumulation of pH-sensitive liposomes. Drug Deliv. Transl. Res. 9, 123-130 (2019).
75. Liu, X. et al. Serum apolipoprotein A-I depletion is causative to silica nanoparticles–induced cardiovascular damage. Proc. Natl. Acad. Sci. U. S. A. 118, e2108131118 (2021).
76. Assali, A. et al. The bio-interface between functionalized Au NR@GO nanoplatforms with protein corona and their impact on delivery and release system. Colloids Surf., B 173, 891-898 (2019).
77. Ghazaryan, A., Landfester, K. & Mailaender, V. Protein deglycosylation can drastically affect the cellular uptake. Nanoscale 11, 10727-10737 (2019).
78. Alnasser, F. et al. Graphene Nanoflake Uptake Mediated by Scavenger Receptors. Nano Lett. 19, 1260-1268 (2019).
79. Soo Choi, H. et al. Renal clearance of quantum dots. Nat. Biotechnol. 25, 1165-1170 (2007).
80. Longmire, M., Choyke, P.L. & Kobayashi, H. Clearance properties of nano-sized particles and molecules as imaging agents: considerations and caveats. Nanomed. 3,

703-717 (2008).
81. Bose, T., Latawiec, D., Mondal, P.P. & Mandal, S. Overview of nano-drugs characteristics for clinical application: the journey from the entry to the exit point. J. Nanopart. Res. 16, 2527 (2014).
82. Chen, L.-T. & Weiss, L. The Role of the Sinus Wall in the Passage of Erythrocytes Through the Spleen. Blood 41, 529-537 (1973).
83. Petros, R.A. & DeSimone, J.M. Strategies in the design of nanoparticles for therapeutic applications. Nat. Rev. Drug Discov. 9, 615-627 (2010).
84. Blanco, E., Shen, H. & Ferrari, M. Principles of nanoparticle design for overcoming biological barriers to drug delivery. Nat. Biotechnol. 33, 941-951 (2015).
