# Supplementary material for: Nanoparticle elasticity affects systemic circulation lifetime by modulating adsorption of apolipoprotein A-I in corona formation
Source: Nat Commun. 2022 Jul 16;13:4137. doi: 10.1038/s41467-022-31882-4 (PMC9288426; doi:10.1038/s41467-022-31882-4)
Supplement: Supplementary file 3 — Reporting Summary [file 41467_2022_31882_MOESM3_ESM.pdf]

nature portfolio

Corresponding author(s): Lihua Yang

Last updated by author(s): Jul 4, 2022

Reporting Summary

Nature Portfolio wishes to improve the reproducibility of the work that we publish. This form provides structure for consistency and transparency in reporting. For further information on Nature Portfolio policies, see our Editorial Policies and the Editorial Policy Checklist.

Statistics

For all statistical analyses, confirm that the following items are present in the figure legend, table legend, main text, or Methods section.

n/a	Confirmed	
☐	☒	The exact sample size (*n*) for each experimental group/condition, given as a discrete number and unit of measurement
☐	☒	A statement on whether measurements were taken from distinct samples or whether the same sample was measured repeatedly
☐	☒	The statistical test(s) used AND whether they are one- or two-sided<br>*Only common tests should be described solely by name; describe more complex techniques in the Methods section.*
☒	☐	A description of all covariates tested
☒	☐	A description of any assumptions or corrections, such as tests of normality and adjustment for multiple comparisons
☐	☒	A full description of the statistical parameters including central tendency (e.g. means) or other basic estimates (e.g. regression coefficient) AND variation (e.g. standard deviation) or associated estimates of uncertainty (e.g. confidence intervals)
☒	☐	For null hypothesis testing, the test statistic (e.g. *F*, *t*, *r*) with confidence intervals, effect sizes, degrees of freedom and *P* value noted<br>*Give P values as exact values whenever suitable.*
☒	☐	For Bayesian analysis, information on the choice of priors and Markov chain Monte Carlo settings
☒	☐	For hierarchical and complex designs, identification of the appropriate level for tests and full reporting of outcomes
☐	☒	Estimates of effect sizes (e.g. Cohen's *d*, Pearson's *r*), indicating how they were calculated

Our web collection on statistics for biologists contains articles on many of the points above.

Software and code

Policy information about availability of computer code

Data collection	Mass spectrometry (MS) instrument-control software Thermo Xcalibur (version: 4.0)
Data analysis	Origin 8.0, Excel 2016, Thermo Proteome Discoverer software suite (version: 2.2.0.388), PKSolver (a freely available menu-driven add-in program for Microsoft Excel)

For manuscripts utilizing custom algorithms or software that are central to the research but not yet described in published literature, software must be made available to editors and reviewers. We strongly encourage code deposition in a community repository (e.g. GitHub). See the Nature Portfolio guidelines for submitting code & software for further information.

Data

Policy information about availability of data

All manuscripts must include a data availability statement. This statement should provide the following information, where applicable:

- Accession codes, unique identifiers, or web links for publicly available datasets
- A description of any restrictions on data availability
- For clinical datasets or third party data, please ensure that the statement adheres to our policy

The authors declare that data supporting the findings of this study are available within the paper and its Supplementary Information files. Source data are provided with this paper. Source data are available for Figure 1e, 2b-c, 2e-i, 2k, 3a-h, 3j, 4a-d, 4f, 5d-i and Supplementary Figure 2b, 3, 4b-c, 7-8, 10-13, 15-17, 18b-c, 19-20 in the associated source data file. The mass spectrometry proteomics data have been deposited to the ProteomeXchange Consortium (http://proteomecentral.proteomexchange.org) via the iProX partner repository [1] with the dataset identifier PXD034004.

[1] Ma J, et al. (2019) iProX: an integrated proteome resource. Nucleic Acids Res, 47, D1211-D1217.

Field-specific reporting

Please select the one below that is the best fit for your research. If you are not sure, read the appropriate sections before making your selection.

- [x] Life sciences
- [ ] Behavioural & social sciences
- [ ] Ecological, evolutionary & environmental sciences

For a reference copy of the document with all sections, see nature.com/documents/nr-reporting-summary-flat.pdf

Life sciences study design

All studies must disclose on these points even when the disclosure is negative.

Sample size	Group/sample sizes were chosen accordingly to previous reports using the same methods. For in vitro cell model and analysis, the reference we referred to is: Schöttler, S. et al. Protein adsorption is required for stealth effect of poly(ethylene glycol)- and poly(phosphoester)-coated nanocarriers. Nat. Nanotechnol. 11, 372-377 (2016). For in vivo blood circulation, the reference we referred to is: Bertrand, N. et al. Mechanistic understanding of in vivo protein corona formation on polymeric nanoparticles and impact on pharmacokinetics. Nat. Commun. 8, 777-784 (2017). Sample sizes for in vitro cell culture and other experiments are indicated in each legend.
Data exclusions	No data were excluded from the analysis; all analyses were performed as described in the Materials and Methods.
Replication	To verify the reproducibility of the cellular assays, each cell study trial was carried out in triplicate and the reported results are the average of twoindependent trials. The observed results indicated that all the attempts at replication were successful.<br>To verify the reproducibility of the animal experiments, mice in each in vivo assay were randomly allocated into different treatment groups with 5 or 6 mice in each group. The observed results suggested that all the attempts at replication were successful.<br>For all proteomics analyses, two independent trials were carried out for each sample. The observed results suggested that all the attempts at replication were successful.
Randomization	In each independent assay involving cell culture, the cell cultures were randomly allocated into the control and treatment groups. To ensure randomization, we collected cell cultures after they have reached appropriate state of cell growth, re-dispersed the as-collected cells as plankton into the expected medium (buffer or nutrient medium), and then inoculated the expected and equal amount (usually measured by volume) of the resulting cell dispersion into each well of a microplate, and the as-inoculated wells were then allocated to the control and different treatment groups.<br>In animal experiments, animals were randomly divided into several groups before any treatment, to ensure the randomness of each group of animals.
Blinding	The investigators were not blinded to group allocation during data collection and/ or analysis. In laboratory studies, investigators normally are not blinded to group allocation during data collection and/or analysis, despite that, in clinical studies, investigators are required to be blinded to group allocation during data collection and/or analysis.

Reporting for specific materials, systems and methods

We require information from authors about some types of materials, experimental systems and methods used in many studies. Here, indicate whether each material, system or method listed is relevant to your study. If you are not sure if a list item applies to your research, read the appropriate section before selecting a response.

Materials & experimental systems

n/a	Involved in the study	
	x	Antibodies
	x	Eukaryotic cell lines
x		Palaeontology and archaeology
	x	Animals and other organisms
x		Human research participants
x		Clinical data
x		Dual use research of concern

Methods

n/a	Involved in the study	
x		ChIP-seq
x		Flow cytometry
x		MRI-based neuroimaging

Antibodies

Antibodies used	Rabbit Anti-APOA1 antibody (purchased from Bioss (Beijing, China)) (catalog number: bs-4573R; lot number: AD073151).
Validation	The antibody was used as received from the vendor without further validation. Its manufacturer, Bioss (Beijing, China), has all related information including list of references listed/posted on the manufacture's website (http://www.bioss.com.cn/prolook_03.asp?id=AF08169606021329&pro37=1).

Eukaryotic cell lines

Policy information about cell lines

Cell line source(s)	The cell lines used in this study were murine macrophage Raw 246.7, murine macrophage Ana-1. All eukaryotic cell lines used in this work were purchased from Cell Bank of the Chinese Academy of Sciences (Shanghai, China)
Authentication	All the eukaryotic cell lines were used as received without further authentication
Mycoplasma contamination	The cell lines were not tested or mycoplasma contamination
Commonly misidentified lines (See ICLAC register)	No commonly misidentified lines were involved in this study

Animals and other organisms

Policy information about studies involving animals; ARRIVE guidelines recommended for reporting animal research

Laboratory animals	The animals used in this work were ICR mice (female , 1-8 weeks old). Mice were housed at a temperature of 22-25 ºC and a 12 h/12 h dark/light cycle. We don't have information on humidity of the mouse housing at hand and did not find the related device that can gauge the humidity in the animal center where we animal studies were carried out.
Wild animals	This work did not involve wild animals
Field-collected samples	This work did not involve samples collected from the field
Ethics oversight	The Animal Care and Use Committee at University of Science Technology of China

Note that full information on the approval of the study protocol must also be provided in the manuscript.
